# Incidence, severity, risk factors and outcomes of SARS-CoV-2 reinfections during the Omicron period: a systematic review and meta-analysis

**DOI:** 10.7189/jogh.15.04032

**Published:** 2025-02-07

**Authors:** Durga Kulkarni, Bohee Lee, Nabihah Farhana Ismail, Ahmed Ehsanur Rahman, Julia Spinardi, Moe H Kyaw, Harish Nair

**Affiliations:** 1 Centre for Global Health, Usher Institute, University of Edinburgh, Edinburgh, UK; 2 National Heart & Lung Institute, Imperial College, London, UK; 3 Communicable Disease Control Unit, Public Health Department, Johor Bahru, Johor State, Malaysia; 4 International Centre for Diarrhoeal Diseases Research, Dhaka, Bangladesh; 5 Pfizer, Vaccines, Emerging Markets, New York, New York, USA; 6 School of Public Health, Nanjing Medical University, Jiangsu, China; 7 MRC/Wits Rural Public Health and Health Transitions Research Unit (Agincourt), School of Public Health, Faculty of Health Sciences, University of the Witwatersrand, Johannesburg, South Africa

## Abstract

**Background:**

Our previous systematic review estimated the cumulative incidence of SARS-CoV-2 reinfections as 1.16% (95% CI = 1.01–1.33%) during the pre-Omicron period. The Omicron variant that emerged in November 2021 was significantly genetically distinct from the previous SARS-CoV-2 variants and thus, more transmissible and posed an increased risk of SARS-CoV-2 reinfections in the population. We, therefore, conducted a fresh systematic review and meta-analysis to estimate the SARS-CoV-2 reinfection burden during the Omicron period.

**Methods:**

We searched CINAHL, Medline, Global Health, Embase, and WHO COVID-19 in October 2023 for studies reporting the SARS-CoV-2 reinfection incidence during the Omicron period. The quality of the included studies was assessed using the Joanna Briggs Institute checklists. Random effects meta-analyses were conducted to estimate the incidence, and requirement of hospitalisation of SARS-CoV-2 reinfections. Symptomatic severity of reinfections and case fatality rates were analysed narratively.

**Results:**

Thirty-six studies were included. The reinfection cumulative incidence during the Omicron period was 3.35% (95% CI = 1.95–5.72%) based on data from 28 studies. The cumulative incidence was higher in 18–59-year-old adults (6.62% (95% CI = 3.22–13.12%)) compared to other age groups and in health care workers (9.88% (95% CI = 5.18–18.03%)) compared to the general population (2.48% (95% CI = 1.34–4.54%)). We estimated about 1.81% (95% CI = 0.18–15.87%) of the reinfected cases required hospitalisation based on limited and highly variable data.

**Conclusions:**

There was an increased risk of reinfections during the Omicron period compared to the pre-Omicron period. The incidence was higher in 18–59-year-old adults and health care workers and generally less severe during the Omicron period. However, data were limited on disease severity and long-term outcomes.

**Registration:**

PROSPERO: CRD42023482598.

SARS-CoV-2 reinfections that seemed rare during the early pandemic period became increasingly frequent with the emergence of new variants [1]. In a previously published systematic review and meta-analysis, we estimated the SARS-CoV-2 reinfection cumulative incidence during the pre-Omicron period as 1.16% (95% CI = 1.01–1.33%) [2]. The incidence, risk and severity of reinfections during the Omicron period have not been systematically summarised.

The Omicron variant was first identified in November 2021 [3]. The World Health Organization designated Omicron (B.1.1.529) as a variant of concern on 26 November 2021 [4]. Recent Omicron subvariants like the BQ.1 and XBB, due to their critical spike mutations, limited the effectiveness of available vaccines and therapeutic monoclonal antibodies in preventing infections [5]. Additionally, previous infections tended to be less effective in preventing reinfections with Omicron compared to the Delta variant [6]. This ability of the Omicron subvariants to escape immune response and relaxation of non-pharmacological interventions (NPIs), cumulatively increased the reinfection and breakthrough infection risk in the population. Hence, conducting continued monitoring and surveillance of SARS-CoV-2 infections and reinfections becomes necessary to identify risks and inform public health measures, including vaccination strategies.

In this systematic review and meta-analysis, we aimed to estimate the global SARS-CoV-2 reinfection burden during the Omicron period. More specifically, this research primarily aimed to estimate the SARS-CoV-2 reinfection incidence in the Omicron period. The secondary aims of this research were to explore risk factors for reinfections by estimating the SARS-CoV-2 reinfection incidence by country-income, age group, vaccination status, sex, population groups, and primary infection variant; the clinical severity of SARS-CoV-2 reinfections including the percentage of cases requiring hospitalisation and the case fatality rate (CFR); and the relationship between SARS-CoV-2 reinfections and long COVID symptoms during the Omicron period.

## METHODS

This systematic review was conducted following the Preferred Reporting Items for Systematic Review and Meta-analysis Protocols (PRISMA-P 2020) guidelines [7]. A protocol was registered on PROSPERO (CRD42023482598). No formal ethical approval was sought as we used only published data.

We searched five databases between 6 and 8 October 2023 – CINAHL, Ovid Medline, Ovid Global Health, Ovid Embase, and the WHO COVID-19 research database (Text S1 in the **Online Supplementary Document**). We used a broad search strategy containing terms related to COVID-19 and reinfections by adapting the search strategies from our previously published study [2].

Reinfection was defined as a primary infection determined by a positive PCR or antigen test for SARS-CoV-2 and a second positive PCR or antigen test at least 45 days from the primary infection. We included prospective and retrospective observational studies, intervention studies, and population or facility-based studies. We searched for studies published from 1 November 2021 onwards, as the Omicron variant was first isolated in November 2021. We did not set any geographical or language restrictions in our searches but excluded studies published in languages other than English. The selection criteria are summarised in Table S1 in the **Online Supplementary Document**.

Two reviewers independently screened, using Covidence, the titles and abstracts of the retrieved studies followed by the full texts of the studies selected as potentially eligible by at least one reviewer [8]. We adapted the data extraction form used for extracting data for our aforementioned systematic review [2]. Two reviewers independently conducted data extraction and assessed the risk of bias in included studies using the Joanna Briggs Critical Appraisal tools. Studies with a score of ≤5 were graded to be of poor quality. Any disagreements were resolved by discussion within the review team.

### Statistical analysis

Statistical analyses were conducted in RStudio. We adopted the random effects meta-analysis model to estimate the incidence and requirement of hospitalisation.

We estimated the SARS-CoV-2 reinfection cumulative incidence during the Omicron period, wherein the primary infections were not restricted to a particular wave (period of dominance of a specific variant). We then reported the estimates by excluding studies of poor quality (studies with a score of ≤5). We also conducted a sensitivity analysis by limiting studies that defined the minimum interval between the two infections as 90 days. Additionally, we conducted subgroup analyses, using available data, to estimate the cumulative incidence by country income (high-income countries (HICs), upper-middle-income countries (UMICs), and lower-middle income countries (LMICs)); population group (health care workers or general population); age group (<18 years, 18–59 years, ≥60 years); COVID vaccination status (<2 doses, ≥2 doses), sex, and primary infection variant (Omicron, Alpha, Delta, and Gamma). Lastly, a sensitivity analysis was undertaken to estimate the cumulative incidence of SARS-CoV-2 reinfections by including data from studies employing only PCR.

Further, we estimated the incidence rate per 10 000 person-days of SARS-CoV-2 reinfections during the Omicron period.

We did not conduct meta-analyses to synthesise severity outcomes, as limited studies reported relevant data (n = 4 for asymptomatic presentation; n = 5 for severe disease). Additionally, the definitions of disease severity were either unclear or non-standardised, and the testing policies were unclear. Similarly, there were limited and heterogeneous data on COVID-19 reinfection case fatality rate (CFR), and these findings were also synthesised narratively.

Lastly, we summarised the range of mean and median intervals between the two infection episodes using available data and according to the primary infection variant.

## RESULTS

**Figure 1:**
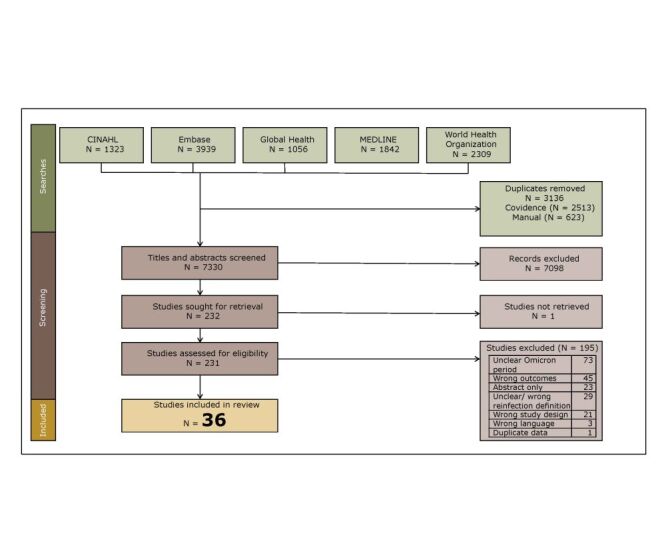
PRISMA flowchart.

This systematic review included 36 studies (**Figure 1**) [9–44]. List of studies excluded at the full-text screening stage with the reason for exclusion is presented in Table S2 in the **Online Supplementary Document**.

### Study characteristics

Twenty-two studies were conducted in HICs, 13 in UMICs and, one in a LMIC. Sixteen studies relied on PCR only, and 20 studies utilised PCR or antigen tests, including rapid diagnostic tests for testing SARS-CoV-2 primary infections and reinfections. Most studies (n = 30) defined the minimum interval between the primary infections and reinfections as 90 days. Of the remaining six studies, two studies used 60 days, and four studies used 45 days as the minimum interval between the two SARS-CoV-2 infections. Two studies focussed on children or adolescents, and five studies on adults. The remaining 29 studies included the general population with people of all ages. Five studies were conducted among health care workers and 28 among the general population. One study each reported data on sumo wrestlers, kidney transplant patients and convalescent plasma donors. All study participants were completely vaccinated in four studies, the vaccination status of the study population was mixed in 30 studies and unclear in two studies.

### Cumulative incidence and risk factors

The pooled cumulative incidence of SARS-CoV-2 reinfections was 3.35% (95% CI = 1.95–5.72%) based on data from 28 studies and with primary infections that were not restricted to a particular period i.e. occurred across any various pre-Omicron and Omicron waves of the COVID-19 pandemic. The lowest estimates (660 / 447 223) were reported in a study from South Korea focussing on adults (between January 2020 and February 2022 and with exclusive PCR testing) [21]. The highest estimates (1007 / 3545) were reported in a study on health care workers from India (between May 2021 and February 2022 and with PCR/antigen testing) [29] (Figure S1 in the **Online Supplementary Document**). When the analysis was limited to good-quality studies (n = 14), the pooled cumulative incidence was 2.59% (95% CI = 1.06–6.20%) (**Table 1**).

**Table 1:** Risk of bias assessment of included studies

Study ID	Author	Were the two groups similar and recruited from the same population?	Were the exposures measured similarly to assign people to both exposed and unexposed groups?	Was the exposure measured in a valid and reliable way?	Were confounding factors identified?	Were strategies to deal with confounding factors stated?	Were the groups/ participants free of the outcome at the start of the study (or at the moment of exposure)?	Were the outcomes measured in a valid and reliable way?	Was the follow up time reported and sufficient to be long enough for outcomes to occur?	Was follow up complete, and if not, were the reasons of loss to follow up described and explored?	Were strategies to address incomplete follow up utilized?	Was appropriate statistical analysis used?	Total score
47	Abuhasira, 2023	1	0	1	1	1	0	1	0	0	0	1	6
2360	Acuna-Castillo, 2023	1	1	1	0	0	0	1	0	0	0	1	5
2649	Banjongjit, 2022	1	1	1	1	1	0	1	0	0	0	1	7
346	Cai, 2023	1	0	1	0	1	0	1	0	0	0	1	5
2945	Cauchi, 2022	1	1	1	0	0	0	1		1	1	1	7
7855	Cegolon, 2023	1	1	1	0	1	1	1	0	0	0	1	7
403	Chemaitelly, 2022	1	1	1	1	1	0	1	0	0	0	1	7
405	Chemaitelly, 2022	1	0	1	1	1	0	1	0	0	0	1	6
3083	Ciuffreda, 2023	1	1	1	1	0	0	1	0	0	0	1	6
460	Cohen, 2023	1	0	1	0	0	0	1	0	0	0	1	4
643	Eythorsson, 2022	1	1	1	0	0	0	1	0	0	0	1	5
702	Freire-Neto, 2022	1	1	1	0	1	0	1	0	0	0	1	6
3610	Gim, 2023	1	1	1	1	1	0	1	0	0	0	1	7
794	Guedes, 2023	1	1	1	1	0	0	1	0	0	0	1	6
3955	Jang, 2022	1	0	1	1	1	0	1	0	0	0	1	6
965	Jang, 2023	1	0	1	1	1	0	1	0	0	0	1	6
4083	Keeling, 2023	1	0	1	0	0	0	1	0	0	0	1	4
1076	Koga, 2022	1	0	1	1	1	0	1	0	0	0	1	6
8491	Lee, 2023	1	0	1	1	1	0	1	0	0	0	1	6
4468	Ma, 2023	0	0	1	0	0	0	1	0	0	0	1	3
1280	Malhotra, 2022	1	0	1	1	1	0	1	1	0	0	1	7
1330	Medic, 2023	1	0	1	1	1	0	1	1	0	0	1	7
1434	Nagao, 2023	1	0	1	1	1	0	1	0	0	0	1	6
1451	Nguyen, 2022	1	1	1	0	0	0	1	0	0	0	1	5
8753	Nguyen, 2023	1	1	1	0	0	0	1	0	0	0	1	5
4880	Ntziora, 2022	1	1	1	0	0	0	1	0	1	0	1	6
1484	Ochoa-Hein, 2022	1	0	1	0	0	0	1	0	0	0	1	4
8803	Ozudogru, 2023	1	1	1	0	0	0	1	0	0	0	1	5
5093	Petrone, 2023	1	1	1	0	0	0	1	0	0	0	1	5
1599	Piazza, 2022	1	0	1	1	0	0	1	0	0	0	1	5
5335	Rothberg, 2023	1	1	1	0	0	0	1	0	0	0	1	5
1743	Sacco, 2022	1	1	1	0	0	0	1	0	0	0	1	5
7383	Spicer, 2023	1	0	1	0	0	0	1	0	0	0	1	4
2093	Vicentini, 2023	1	0	1	1	0	0	1	0	0	0	1	5
6147	Ye, 2023	1	0	1	0	1		1	1	0	0	1	6
6165	Yu, 2023	1	0	1	0	1	0	1	1	0	0	1	6

The reinfection cumulative incidence was 4.29% (95% CI = 2.39–7.59%) when the analysis was limited to studies (n = 22) that defined the minimum interval between two infections as 90 days.

Overall, the highest cumulative incidence estimates were reported from a study conducted in an LMIC (28.41% (95% CI = 26.95–29.91%)) followed by those conducted in HICs (4.85% (95% CI = 2.83–8.190%)) and UMICs (1.53% (95% CI = 0.61–3.76%)). The pooled reinfection cumulative incidence was higher in studies (n = 5) focussing on health care workers (9.88% (95% CI = 5.18–18.03%)) than in those (n = 21) conducted in the general population (2.48% (95% CI = 1.34–4.54%)). It was 6.62% (95% CI = 3.22–13.12%) in adults aged 18–59 years. In those aged <18 years and ≥60 years, it was 1.89% (95% CI = 1.10–3.24%) and 2.79% (95% CI = 1.40–5.50%) respectively. The overall reinfection cumulative incidence for all ages, from analysis limited to studies reporting age-stratified data, was 3.60% (95% CI = 2.32–5.53%).

When studies reported reinfection cumulative incidence estimates stratified by vaccination status (n = 8), the pooled cumulative incidence was 11.00% (95% CI = 5.06–22.27%) in those with incomplete vaccination (zero or single dose) and 6.75% (95% CI = 2.48–17.12%) in those with complete vaccination (at least 2 doses). The overall reinfection cumulative incidence (irrespective of vaccination status) in these studies was 8.64% (95% CI = 4.59–15.69%).

The pooled cumulative incidence was slightly higher in females (6.39% (95% CI = 2.49–15.40%)) than in males (5.78% (95% CI = 2.18–14.45%)). However, the difference was statistically insignificant. The overall cumulative incidence (both sexes) in the studies reporting sex-stratified data was 6.35% (95% CI = 2.43–15.62%) (n = 7). Only three studies reported the cumulative incidence estimates by comorbidity status. The estimates were generally lower in the groups with a comorbidity than in the entire population (Table S3 in the **Online Supplementary Document**). Exceptions to this finding were in the study by Abuhasira et al. that reported higher cumulative incidence estimates in the asthma group (9.23% (95% CI = 9.08–9.38%)) and the immunocompromised group (9.1% (95% CI = 8.88–9.40%)) than the entire study population (8.70% (95% CI = 8.65–8.75%) [9].

When studies reported data stratified by SARS-CoV-2 primary infection variant or limited to a particular SARS-CoV-2 wave, the pooled cumulative incidence was 4.89% (95% CI = 1.87–12.21%) when the primary infection variant was Omicron (or during the Omicron period) (n = 9). It was 2.12% (95% CI = 1.76–2.55%) when the primary infection occurred during the Alpha dominance period (n = 1); 1.08% (95% CI = 0.67–1.74%) when the primary infection occurred during the Delta dominance period (n = 3); and 1.01% (95% CI = 0.86–1.19%) when the primary infection occurred in Gamma dominance period (n = 1).

When the analyses were restricted to PCR-only studies (n = 12), the pooled SARS-CoV-2 reinfection cumulative incidence during the Omicron period was 2.21% (95% CI = 1.02–4.72%).

**Figure 2:**
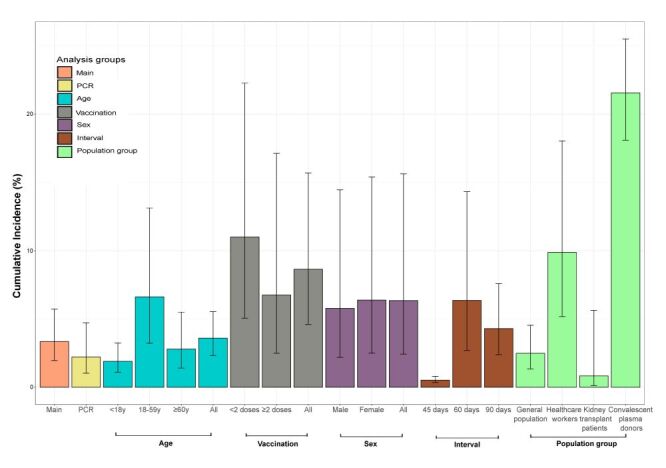
Summary of findings – cumulative incidence of SARS-CoV-2 reinfections during the Omicron period.

The cumulative incidence and risk factors analysis findings are summarised in **Figure 2****.**

### Incidence rate

The SARS-CoV-2 reinfection pooled incidence rate was 7.50 (95% CI = 2.28–12.70) per 10 000 person-days in studies conducted in the general population (Figure S2 in the **Online Supplementary Document**). In two studies conducted on health care workers, the reported incidence rates were 4.93 (95% CI = 4.62–5.23) per 10 000 person-days [14] and 45.6 (95% CI = 42.9–48.5) per 10 000 person-days [29].

Three studies reported the Omicron reinfection incidence rate stratified by the primary infection variant. The incidence rate was lower for Omicron reinfections when the primary infections were also from the Omicron period (3 (95% CI = 2–3) per 10 000 person-days) than when the primary infections were from the pre-Omicron period (14 (95% CI = 13–15) per 10 000 person-days) as demonstrated in one study from Italy [14]. Another study from Chile reported a significantly lower incidence of reinfections than others, throughout their study period. The reinfection incidence rate was highest (0.21 (95% CI = 0.17–0.25) per 10 000 person-days) when primary infection occurred during the gamma dominant period and reinfections between 25 December 2021 and 10 April 2022 (i.e. early Omicron period). In this study, the reinfection rate during the Omicron period was lowest (0.02 (95% CI = 0.01–0.02) per 10 000 person-days) when primary infection occurred during the early pandemic period and reinfections between 2 May 2022 and 31 July 2022 (later omicron period) [10]. Yu et al., in their study from China, reported an incidence rate of 6.6 per 10 000 person-days when the primary infections occurred during the Omicron period [44].

### Disease severity

Only four studies reported data on asymptomatic reinfection cases (Table S4 in the **Online Supplementary Document**). The percentage of reinfections presenting with asymptomatic presentation varied greatly in these studies (range = 4.36–92.5%). In the four studies that reported data on severe reinfection cases, the percentage of reinfections presenting with severe disease ranged from 0.05 to 6.08% [16,40,42,44].

Based on data from five studies, 1.81% (95% CI = 0.18–15.87%) of reinfection cases required hospitalisation (43 677 / 2 815 300) [9,20,28,30,39] (Figure S3 in the **Online Supplementary Document**).

Between 0.05 and 6.08% of reinfection cases progressed to severe disease. Of the four studies that reported these data (Table S5 in the **Online Supplementary Document**), the highest and lowest estimates were reported in the general population in a study in China (9 / 148) [44] and Qatar (4 / 7995) [16], respectively. No individuals with reinfections required admission to the intensive care unit in a study following up 343 cases of reinfections [16]. In another study, 41 out of 1467 individuals with reinfections required ICU admission [39].

### Case fatality rate

Only five studies reported data on survival outcomes in individuals with SARS-CoV-2 reinfections during the Omicron period [9,16,27,28,42]. There were 6723 deaths among 2 894 995 reinfection cases in total (Table S6 in the **Online Supplementary Document**). The highest CFR (0.34%) was reported in a study from Qatar among the general population and with a sample of 7995 reinfection cases [16]. However, none of the deaths were related to COVID-19. The lowest estimate was reported in a study conducted in Italy among the general population, with one death reported in a sample of 3243 reinfection cases. In this study, death was attributed to a SARS-CoV-2 infection if occurring within 90 days from diagnosis and fulfilled the criteria for reporting COVID-19 as the main cause of death [42].

### Interval between primary infections and reinfections

The mean or median interval between primary infections and reinfections was reported in 10 studies. The mean interval ranged between 167 and 411 days and the median interval between 127 and 507 days when both, primary infections and reinfections, occurred during the Omicron period. The mean interval was higher when the primary infections occurred during the pre-Omicron period and highest for early strains – Omicron reinfections (range = 611 to 736 days) (**Table 2**).

**Table 2:** Mean and median interval between primary infections and reinfections

Primary infection strain	Summary measure for the interval between primary infection and reinfection	Range of summary measure (in days)	Number of studies, n
Pre-Omicron	x̄	512	1 [14]
Others (early strains)	x̄	611 (wave 4)* and 736 (wave 5)†	1 [10]
Gamma	x̄	310 (wave 4)* and 432 (wave 5)†	1 [10]
Delta	x̄	128 (wave 4)* and 219 (wave 5)†	1 [10]
Omicron	x̄	167 to 411	2 [28,44]
Omicron	MD	127 to 507	3 [10,14,22]
Any	x̄	287 to 507	5 [14,19,22,36,39]
Any	MD	155 to 504	2 [18,41]

MD – median, x̄ – mean

*Wave 4 = period between 25 December 2021 and 10 April 2022.

†Wave 5 = period between 2 May 2022 and 31 July 2022

## DISCUSSION

This is the most recent and comprehensive global review estimating the reinfection burden during the Omicron period. The SARS-CoV-2 reinfection incidence during the Omicron period was 3.35% (95% CI = 1.95–5.72%). The reinfection incidence was higher in the 18–59 years age group, health care workers, and those with ≤1 vaccine dose than those aged <18 years or >60 years, the general population and those with ≥2 vaccine doses respectively. Our findings suggest that 1.81% (95% CI = 0.18–15.87%) of reinfections during the Omicron period required hospitalisation. However, data were limited on disease severity and post-infection outcomes.

The reinfection cumulative incidence during the pre-Omicron period estimated in our previous systematic review that included PCR-only studies (1.16% (95% CI = 1.01–1.33%)) is slightly lower than our current estimate with PCR-only studies (2.21% (95% CI = 1.02–4.72%)) and significantly lower than the estimate including PCR or antigen tests (3.35% (95% CI = 1.95–5.72)) [2]. This finding is similar to findings from other studies showing higher reinfection risk during Omicron dominance than previous waves [45,46]. It is still important to highlight that very few studies conducted genomic testing to determine the variant associated with reinfections [17,26,32,33,37,40]. In the remaining studies, genomic testing was not undertaken, and the variant associated with reinfections may or may not have been Omicron, although the infection occurred during the Omicron period. Additionally, the follow-up period of studies may have impacted the reinfection rates such that studies with longer follow-up periods are likely to have higher reinfection rates, and the follow-up period of included studies ranged between three and 36 months.

The SARS-CoV-2 reinfection cumulative incidence was slightly lower in studies employing PCR testing (2.21% (95% CI = 1.02–4.72%)) than those with either PCR or antigen tests (3.35% (95% CI = 1.95–5.72%)). This finding is likely driven by two factors operating in opposite directions- the sensitivity of tests and access to testing. PCR tests are considered gold-standard and more sensitive than antigen tests, especially in infections with low viral loads [47]. However, PCR testing requires specialised laboratory equipment and a trained workforce, limiting their accessibility [48]. Antigen tests, however, are advantageous in terms of speed, cost, and accessibility, particularly in resource-constrained settings that can be expected to result in enhanced testing coverage [49].

The reinfection cumulative incidence during the Omicron period was higher in adults aged 18–59 years than those aged <18 years or ≥60 years, and the adjusted hazard/odds/risk ratio estimates for age also demonstrated similar patterns (Table S7a in the **Online Supplementary Document**). Although data in the included studies were non-standardised, the working-age population were shown to be at a higher reinfection risk than the paediatric or older adult populations. This finding is similar to our finding for reinfections in the pre-Omicron period and could be a result of the participation of this population in income-generating activities with greater social interactions and travel, leading to increased exposure to the virus [2]. Our data highlight the importance of improving vaccination coverage in the non-elderly age groups to control viral transmission in the population.

The reinfection risk was lower in individuals with ≥2 vaccine doses than those with ≤1 dose and lower in most studies in vaccinated groups than in unvaccinated groups (Table S7b in the **Online Supplementary Document**). Data were, however, limited as only eight studies reported cumulative incidence stratified by vaccination status. Eight studies also reported the adjusted or unadjusted hazard/odds/risk ratios of reinfections according to vaccination status. Data showed that vaccination with ≥2 doses had a protective value against reinfections than zero vaccine doses in adjusted analyses (Table S7b in the **Online Supplementary Document**). A published systematic review showed that vaccination with two doses was more protective against Delta than Omicron infections, but booster dose provided additional protection against Omicron, thus highlighting the importance of booster vaccination [6]. It is important to note that the studies in our systematic review were reported from different regions, with varied data collection periods and with dissimilar vaccination policies, both in terms of type of vaccine and eligibility for vaccination. In addition to the number of doses, the types of vaccine, and their varying efficacy and effectiveness at the population level and in different risk groups, it becomes important to consider the temporality of vaccination and the waning of vaccine immunity over time. Thus, further research is needed that analyses the time from vaccination until reinfection.

Currently, COVID-19 reinfection burden and severity in persons with comorbidities are poorly understood. Data on reinfection incidence according to comorbidity status in the present study were also limited (n = 3). When adjusted for other factors (Table S7c in the **Online Supplementary Document**), the hazard ratios for reinfections were reportedly higher in populations living with asthma [9], chronic kidney failure [9], hypertension [9], ischaemic heart disease [9], history of stroke or transient ischaemic attack [9], and immunosuppression [9,23]. In the study by Jang et al., the adjusted hazard ratios for reinfections were also significantly higher for the populations living in long-term care facilities than the general population (2.34% (95% CI = 2.26–2.43%)) where one would expect to find a higher proportion of people with underlying health conditions [9,23]. It is, therefore, essential that further research continues to be undertaken to explore the necessity of vaccination policies targeting these at-risk population groups. This becomes even more important, especially in the post-pandemic period when NPIs have been withdrawn, the level of shielding measures can be expected to have been relaxed, and there is an increased likelihood of social interactions that can increase the reinfection risk in these populations.

The reinfection incidence rate during the Omicron period was higher when primary infections occurred during the pre-Omicron period (14 (95% CI = 13–15) per 10 000 person-days) than the Omicron period (3 (95% CI = 2–3) per 10 000 person-days) [14]. Conversely, we observed that the Omicron reinfection cumulative incidence was higher after primary Omicron infections (4.89% (95% CI = 1.87–12.21%)) than after primary Delta (1.08% (95% CI = 0.67–1.74%)), Gamma (1.01% (95% CI = 0.86–1.19%)), or Alpha (2.12% (95% CI = 1.76–2.55%)) infections. These findings must be interpreted cautiously as all studies did not report a laboratory confirmation of the variant causing infection and assumed that the variant causing infection was the most dominant variant circulating in their region at the given time. Moreover, data were limited, and only one study each, reported on Gamma-Omicron and Alpha-Omicron reinfections. The role of prior infection caused by various variants on the durability of immunity to subsequent infection with Omicron sub-lineage is not established.

The high variability in the percentage of asymptomatic reinfections across studies could be attributed to the differences in definitions used for the severity of infections (less likely) and more likely due to differences in testing strategies (the severity definitions and testing strategies were unclear in most studies). Asymptomatic infections and mild cases are less likely to undergo testing. Literature suggests that Omicron infections tend to have a milder presentation than infections by the earlier variants [50]. Our incidence estimates, therefore, are underestimates as we believe that a significant number of asymptomatic or mild reinfections may have gone undetected, especially as contact tracing and testing strategies became increasingly lax during the Omicron period than the early pandemic period [51]. Only five studies provided data on hospitalisation, without any information on the criteria for hospitalisation. This estimate (1.81% (95% CI = 0.18–15.87%)) is lower than the hospitalisation estimate reported during the pre-Omicron period (8.12% (95% CI = 5.30–11.39%)), but the confidence intervals overlap [2]. Although some studies in the pre-Omicron period have shown that reinfections increase the hospitalisation risk, our findings are not surprising given the nature of Omicron infections. As previously mentioned, Omicron infections tend to be less severe than earlier variants [50]. Second, the hospitalisation criteria were eased over time in most countries owing to this less severe presentation of Omicron infections and also as infection control measures (for example, isolation or contact tracing) became less stringent. Lastly, more individuals would have been vaccinated during the Omicron period than earlier in the pandemic. This could be an additional factor in reducing hospitalisation risks in vaccinated individuals, as studies have shown that SARS-CoV-2 infections in vaccinated individuals were associated with a lower hospitalisation risk than infections in unvaccinated individuals [52]. It remains unclear if certain groups (for example, older adults or those living with comorbidities) are at an increased hospitalisation risk after reinfections. The CFR reported in studies (n = 5) varied between 0.00% and 0.34%. However, the sources of mortality data and the definition of COVID-attributable deaths (including the follow-up periods) were variable across the studies. In the study reporting the highest CFR, none of the deaths were COVID-19-attributable (Table S6 in the **Online Supplementary Document**). The CFR during the pre-Omicron period was reportedly higher (0.71%), but these also included non-COVID-19-attributable deaths [2]. Although research suggests that Omicron reinfections tend to be less severe, none of the studies eligible for inclusion in this review reported on long COVID and there were very limited data on reinfection disease severity. As a result, more research on long COVID symptoms and survival outcomes in these patients over time is needed. This may be particularly important in those living with underlying health conditions.

We acknowledge a few additional limitations of this review. The case definitions of reinfections and the testing policies were not standardised across studies. Most studies were conducted in HICs, a few in UMICs and only one in an LMIC which limits the generalisability of the findings and may not represent the true global population. Moreover, it is important to highlight that the included studies had varying follow up periods, population groups, data sources, testing strategies, vaccination policies including the type of vaccines, and were conducted at different times during the Omicron period, and findings should therefore be interpreted with caution. Given the team capacities, we included only English language studies which could have led to language bias.

## CONCLUSIONS

In conclusion, the SARS-CoV-2 reinfection risk during the Omicron period was considerable and higher than that in the pre-Omicron period. Reinfection incidence was lower in completely vaccinated groups compared to those with incomplete vaccination or unvaccinated, thus highlighting the importance of SARS-CoV-2 vaccination. Higher incidence in the working population (age group 18–59 years) also demonstrated the need to vaccinate the non-elderly age groups. However, limited data on reinfection risk in high-risk groups and the disease severity or the requirement of hospitalisations and survival outcomes were available. Data on the occurrence of any long-term or long COVID symptoms after reinfections were particularly lacking. Further research exploring these aspects of reinfections in the Omicron period and of reinfections with new emerging Omicron sub-lineages is needed to help inform public health policy and planning.

## Additional material


Online Supplementary Document
