# Supplementary material for: Incidence, severity, risk factors and outcomes of SARS-CoV-2 reinfections during the Omicron period: a systematic review and meta-analysis
Source: J Glob Health. 2025 Feb 7;15:04032. doi: 10.7189/jogh.15.04032 (PMC11803431; doi:10.7189/jogh.15.04032)

Table of Contents

Text S1: Search strategies 2

Table S1: Eligibility criteria for selection of studies 6

Table S2: List of studies excluded at the full-text review with the reason for exclusion 7

Table S3: Cumulative incidence of SARS-CoV-2 reinfections by comorbidity status 39

Table S4: Data on asymptomatic infections 40

Table S5: Percentage of reinfections progressing to severe disease 40

Table S6: Case fatality rate reported in included studies 41

Table S7a: Age as a risk factor for SARS-CoV-2 reinfections: adjusted and unadjusted hazard ratios, odds ratio and risk ratios reported in included studies 42

Table S7b: Protection by COVID vaccines from SARS-CoV-2 reinfections: adjusted and unadjusted hazard ratios, odds ratios, and risk ratios 44

Table S7c: Comorbidities as a risk of SARS-CoV-2 reinfections: Adjusted and unadjusted hazard ratios 46

Table S7d: Sex as a risk factor of SARS-CoV-2 reinfections: Adjusted and unadjusted hazard ratios, odds ratios and unadjusted risk ratio in females compared to males 47

Figure S1a: Cumulative incidence of SARS-CoV-2 reinfections during the Omicron period (n = 28) 48

Figure S1b: Cumulative incidence of SARS-CoV-2 reinfections during the Omicron period by definition of minimum interval between the two infections (n = 28) 49

Figure S1c: Cumulative incidence of SARS-CoV-2 reinfections by population type (n = 28) 50

Figure S1d: Cumulative incidence of SARS-CoV-2 reinfections by age (n = 16) 51

Figure S1e: Cumulative incidence (incidence proportion) of SARS-CoV-2 reinfections by vaccination status (n = 8) 52

Figure S1f: Cumulative incidence (incidence proportion) of SARS-CoV-2 reinfections by sex (n = 7) 53

Figure S2: Incidence rate of SARS-CoV-2 reinfections per 1000 person days in the general population (n = 3) 54

Figure S3: Percentage of reinfection cases requiring hospital admission 55

Text S1: Search strategies

WHO COVID-19 Research Database (searched on 08 Oct 2023)

(((ti:(reinfect* OR "re-infect" OR "re-infected" OR "re-infection" OR "re-infections")) OR (ab:(reinfect* OR "re-infect" OR "re-infected" OR "re-infection" OR "re-infections")) OR (ti:("repeat infection" OR "repeat infections" OR "repeated infection" OR "repeated infections")) OR (ab:("repeat infection" OR "repeat infections" OR "repeated infection" OR "repeated infections")) OR (ti:("recurring infection" OR "recurrent infection")) OR (ab:("recurring infection" OR "recurrent infection")) OR (ti:("recurrent Covid" OR "recurrent Covid-19" OR "recurrent Sars-Cov-2" OR "recurrent positive" OR "recurrent positives" OR "recurrent positivity")) OR (ab:("recurrent Covid" OR "recurrent Covid-19" OR "recurrent Sars-Cov-2" OR "recurrent positive" OR "recurrent positives" OR "recurrent positivity")) OR (ti:("recurring Covid" OR "recurring Covid-19" OR "recurring Sars-Cov-2" OR "recurring positive" OR "recurring positives" OR "recurring positivity")) OR (ab:("recurring Covid" OR "recurring Covid-19" OR "recurring Sars-Cov-2" OR "recurring positive" OR "recurring positives" OR "recurring positivity")) OR (ti:("reactivate Covid" OR "reactivate Covid-19" OR "reactivate Sars-Cov-2" OR "reactivating Covid" OR "reactivating Covid-19" OR "reactivating Sars-Cov-2" OR "reactivated Covid" OR "reactivated Covid-19" OR "reactivated Sars-Cov-2" OR "reactivation of Covid" OR "reactivation of Covid-19" OR "reactivation of Sars-Cov-2")) OR (ab:("reactivate Covid" OR "reactivate Covid-19" OR "reactivate Sars-Cov-2" OR "reactivating Covid" OR "reactivating Covid-19" OR "reactivating Sars-Cov-2" OR "reactivated Covid" OR "reactivated Covid-19" OR "reactivated Sars-Cov-2" OR "reactivation of Covid" OR "reactivation of Covid-19" OR "reactivation of Sars-Cov-2")) OR (ti:("re-activate Covid" OR "re-activate Covid-19" OR "re-activate Sars-Cov-2" OR "re-activating Covid" OR "re-activating Covid-19" OR "re-activating Sars-Cov-2" OR "re-activated Covid" OR "re-activated Covid-19" OR "re-activated Sars-Cov-2" OR "re-activation of Covid" OR "re-activation of Covid-19" OR "re-activation of Sars-Cov-2")) OR (ab:("re-activate Covid" OR "re-activate Covid-19" OR "re-activate Sars-Cov-2" OR "re-activating Covid" OR "re-activating Covid-19" OR "re-activating Sars-Cov-2" OR "re-activated Covid" OR "re-activated Covid-19" OR "re-activated Sars-Cov-2" OR "re-activation of Covid" OR "re-activation of Covid-19" OR "re-activation of Sars-Cov-2")) OR (ti:(repositiv* OR "re-positive" OR "re-positives" OR "re-positivity" OR "repeated positive" OR "repeated positives" OR "repeat positive" OR "repeat positives" OR "repeat positivity" OR "repeated positivity")) OR (ab:(repositiv* OR "re-positive" OR "re-positives" OR "re-positivity" OR "repeated positive" OR "repeated positives" OR "repeat positive" OR "repeat positives" OR "repeat positivity" OR "repeated positivity"))) AND ((entry_date:2021* OR entry_date:2022* OR entry_date:2023*)))

Filters applied:
Database
MEDLINE
EMBASE
ICTRP
Scopus
EuropePMC
Web of Science
ProQuest Central
GIM
Academic Search Complete
ScienceDirect
CAB Abstracts
PubMed
MDPI
Lanzhou University/CNKI
NCCMT Repository

Centers for Disease Control and Prevention
PMC
Africa Wide Information
WHO COVID
LILACS (Americas)
PubMed Central
Wiley
Indonesian Research
APA PsycInfo
CINAHL
Ichushi
International HTA Db
J-STAGE
NBER
National Technical Information Service
Russian Science Citation Index

Embase (1980 to 2023 Week 39) (searched on 07 Oct 2023)

1	exp Coronavirus infection/ or exp coronavirus disease 2019/
2	exp Severe acute respiratory syndrome coronavirus 2/
3	(Covid or COVID-19* or Coronavirus disease 2019* or Coronavirus 2019* or COVID-2019* or SARS-CoV-2 or SARS CoV 2 or SARS-CoV-2019).ti,ab.
4	1 or 2 or 3
5	exp reinfection/
6	exp recurrent infection/
7	exp recurrent disease/
8	(reinfect* or re-infect*).ti,ab.
9	(repeat infection* or repeated infection*).ti,ab.
10	prior infection.mp.
11	5 or 6 or 7 or 8 or 9 or 10
12	4 and 11
13	(2021* or 2022* or 2023*).em.
14	12 and 13
15	14 not ((exp animal/ or nonhuman/) not exp human/)
16	exp Coronavirus infection/ or exp coronavirus disease 2019/
17	exp Severe acute respiratory syndrome coronavirus 2/
18	(Covid or COVID-19* or Coronavirus disease 2019* or Coronavirus 2019* or COVID-2019* or SARS-CoV-2 or SARS CoV 2 or SARS-CoV-2019).ti,ab.
19	16 or 17 or 18
20	exp reinfection/
21	exp recurrent infection/
22	exp recurrent disease/
23	(reinfect* or re-infect*).ti,ab.
24	(repeat infection* or repeated infection*).ti,ab.
25	prior infection.mp.
26	20 or 21 or 22 or 23 or 24 or 25

27	19 and 26
28	(2021* or 2022* or 2023*).em.
29	27 and 28
30	29 not ((exp animal/ or nonhuman/) not exp human/)

Ovid MEDLINE(R) 1946 to Sep Week 4 2023 (searched on 06 Oct 2023)

1	(Covid or COVID-19* or Coronavirus disease 2019* or Coronavirus 2019* or COVID-2019* or SARS-CoV-2 or SARS CoV 2 or SARS-CoV-2019).ti,ab.
2	exp COVID-19/
3	exp SARS-CoV-2/
4	1 or 2 or 3
5	exp Recurrence/
6	exp Reinfection/
7	(reinfect* or re-infect*).ti,ab.
8	(repeat infection* or repeated infection*).ti,ab.
9	prior infection*.mp.
10	5 or 6 or 7 or 8 or 9
11	4 and 10
12	(2021* or 2022* or 2023*).em.
13	11 and 12
14	exp animals/ not humans.sh.
15	13 not 14

Global Health (searched on 6 Oct 2023)

1	Betacoronavirus/ or Human Coronaviruses/ or exp severe acute respiratory syndrome-related coronavirus/
2	(Covid or COVID-19* or Coronavirus disease 2019* or Coronavirus 2019* or COVID-2019* or SARS-CoV-2 or SARS CoV 2 or SARS-CoV-2019).ti,ab.
3	1 or 2
4	exp reinfection/
5	(reinfect* or re-infect*).ti,ab.
6	(repeat infection* or repeated infection*).ti,ab.
7	prior infection*.mp.
8	4 or 5 or 6 or 7
9	3 and 8
10	("2021" or "2022" or "2023").dp.
11	9 and 10
12	exp animals/
13	humans/
14	12 not 13
15	11 not 14

CINAHL (searched on 6 Oct 2023)

S1	(MH "COVID-19") OR (MH "SARS-CoV-2")	
S2	TI ((COVID OR "COVID-19*" or "Coronavirus disease 2019*" or "Coronavirus 2019*" or "COVID-2019*" or "SARS-CoV-2" or "SARS CoV 2" or "SARS-CoV-2019")) OR AB ((COVID or "COVID-19*" or "Coronavirus disease 2019*" or "Coronavirus 2019*" or "COVID-2019*" or "SARS-CoV-2" or "SARS CoV 2" or "SARS- CoV-2019"))	
S3	S1 OR S2	
S4	(MH "Reinfection")	
S5	(MH "Recurrence")	
S6	TI (reinfect* or "re-infect*") OR AB ((reinfect* or "re-infect*")	
S7	TI ("repeat infection*" or "repeated infection*") OR AB ("repeat infection*" or "repeated infection*")	
S8	TI prior infection* OR AB prior infection*	
S10	S4 OR S5 OR S6 OR S7 OR S8	
S11	S3 AND S10	Limiters - Publication Year: 2021-2023

Table S1: Eligibility criteria for selection of studies

	Include	***Exclude***
Population	Individuals of all ages	Not applicable
Exposure	At least one antigen test or PCR-confirmed SARS-CoV-2 infection (referred to as primary infection in this review)	Individuals without antigen-confirmed or PCR-confirmed SARS-CoV-2 primary infection
Comparator group	Not applicable	Not applicable
Outcome	• *Primary outcome:*<br>SARS-CoV-2 reinfection incidence rate OR SARS-CoV-2 reinfection incidence proportion (where SARS-CoV-2 reinfection is defined as PCR or antigen test confirmed SARS-CoV-2 infection and with the subsequent infection (reinfection) occurring at least 45 days after recovery from the primary SARS-CoV-2 infection)<br>• *Secondary outcomes:*<br>1. Clinical severity of SARS-CoV-2 reinfections<br>2. Outcomes of SARS-CoV-2 reinfections<br>3. Risk factors of SARS-CoV-2 reinfections<br>4. Mean time interval between SARS-CoV-2 primary infection and re-infection<br>5. Association between SARS-CoV-2 reinfections and long COVID symptoms (long COVID as defined by individual study authors)	• SARS-CoV-2 reinfection is not antigen-confirmed or PCR-confirmed<br>• The interval between primary infection and reinfection is not ≥45 days
Context	Studies conducted in any country<br>Studies conducted in any setting (general population, healthcare, educational, workplace, and other clinical<br>Settings)	Not applicable
Publication Type	• Cross-sectional studies<br>• Case-control studies<br>• Cohort studies<br>• Quasi-experimental studies<br>• Randomised control trials	• Case studies, case series, conference abstracts, technical reports, pre-print publications, systematic reviews OR,<br>• Studies that involved fewer than 100 patients with initial infection

Table S2: List of studies excluded at the full-text review with the reason for exclusion

Title	Authors	Published Year	Reason for exclusion
Long-term persistence of RBD+ memory B cells encoding neutralizing antibodies in SARS-CoV-2 infection	Abayasingam, A.; Balachandran, H.; Agapiou, D.; Hammoud, M.; Rodrigo, C.; Keoshkerian, E.; Li, H.; Brasher, N. A.; Christ, D.; Rouet, R.; Burnet, D.; Grubor-Bauk, B.; Rawlinson, W.; Turville, S.; Aggarwal, A.; Stella, A. O.; Fichter, C.; Brilot, F.; Mina, M.; Post, J. J.; Hudson, B.; Gilroy, N.; Dwyer, D.; Sasson, S. C.; Tea, F.; Pilli, D.; Kelleher, A.; Tedla, N.; Lloyd, A. R.; Martinello, M.; Bull, R. A.	2021	wrong outcomes
The prevalence of and factors related to reinfection with COVID-19 in Ahvaz, Iran: A comparative cross-sectional study	Abedi, P.; Afshari, P.; Ansari, S.; Alavi, S. M.; Dashtpayma, S.; Amiri, H.	2023	not Omicron period
SARS-CoV-2 Infection-Blocking Immunity Post Natural Infection: The Role of Vitamin D	Abu Fanne, R.; Moed, M.; Kedem, A.; Lidawi, G.; Maraga, E.; Mohsen, F.; Roguin, A.; Meisel, S. R.	2023	wrong outcomes
Post-COVID-19 syndrome and diabetes mellitus: a propensity-matched analysis of the International HOPE-II COVID-19 Registry	Abumayyaleh, M.; Nunez Gil, I. J.; Viana, LLamas M. C.; Raposeiras Roubin, S.; Romero, R.; Alfonso-Rodriguez, E.; Uribarri, A.; Feltes, G.; Becerra-Munoz, V. M.; Santoro, F.; Pepe, M.; Castro Mejia, A. F.; Signes-Costa, J.; Gonzalez, A.; Marin, F.; Lopez-Pais, J.; Manzone, E.; Vazquez Cancela, O.; Paeres, C. E.; Masjuan, A. L.; Velicki, L.; Weiss, C.; Chipayo, D.; Fernandez-Ortiz, A.; El-Battrawy, I.; Akin, I.	2023	not Omicron period
Assessment of the Risk of Severe Acute Respiratory Syndrome Coronavirus 2 (SARS-CoV-2) Reinfection in an Intense Reexposure Setting	Abu-Raddad, L. J.; Chemaitelly, H.; Malek, J. A.; Ahmed, A. A.; Mohamoud, Y. A.; Younuskunju, S.; Ayoub, H. H.; Al Kanaani, Z.; Al Khal, A.; Al Kuwari, E.; Butt, A. A.; Coyle, P.; Jeremijenko, A.; Kaleeckal, A. H.; Latif, A. N.; Shaik, R. M.; Abdul Rahim, H. F.; Yassine, H. M.; Al Kuwari, M. G.; Al Romaihi, H. E.; Al-Thani, M. H.; Bertollini, R.	2021	not Omicron period

Title	Authors	Published Year	Reason for exclusion
Relative infectiousness of SARS-CoV-2 vaccine breakthrough infections, reinfections, and primary infections	Abu-Raddad, Laith J.; Chemaitelly, Hiam; Ayoub, Houssein H.; Tang, Patrick; Coyle, Peter; Hasan, Mohammad R.; Yassine, Hadi M.; Benslimane, Fatiha M.; Al-Khatib, Hebah A.; Al-Kanaani, Zaina; Al-Kuwari, Einas; Jeremijenko, Andrew; Kaleeckal, Anvar Hassan; Latif, Ali Nizar; Shaik, Riyazuddin Mohammad; Abdul-Rahim, Hanan F.; Nasrallah, Gheyath K.; Al-Kuwari, Mohamed Ghaith; Butt, Adeel A.; Al-Romaihi, Hamad Eid; Al-Khal, Abdullatif; Al-Thani, Mohametabd H.; Bertollini, Roberto	2022	not Omicron period
Association of Prior SARS-CoV-2 Infection With Risk of Breakthrough Infection Following mRNA Vaccination in Qatar	Abu-Raddad, Laith J.; Chemaitelly, Hiam; Ayoub, Houssein H.; Yassine, Hadi M.; Benslimane, Fatiha M.; Al Khatib, Hebah A.; Tang, Patrick; Hasan, Mohammad R.; Coyle, Peter; Al Kanaani, Zaina; Al Kuwari, Einas; Jeremijenko, Andrew; Kaleeckal, Anvar Hassan; Latif, Ali Nizar; Shaik, Riyazuddin Mohammad; Abdul Rahim, Hanan F.; Nasrallah, Gheyath K.; Al Kuwari, Mohamed Ghaith; Butt, Adeel A.; Al Romaihi, Hamad Eid; Al-Thani, Mohamed H.; Al Khal, Abdullatif; Bertollini, Roberto	2021	not Omicron period
Reinfection by SARS-CoV-2: The first one in a family reported in Spain ReinfecciÃ³n por SARS-CoV-2: primer caso en una familia referido en EspaÃ±a	Aguilar-Shea, Antonio L.; GutiÃ©rrez-MartÃn-Arroyo, JoaquÃn Vacas-CÃ³rdoba Miguel Gallardo-Mayo Cristina	2021	wrong study design
Suspected reinfections of SARS-COV-2 in Khyber Pakhtunkhwa, Pakistan - analysis of province-wide testing database	Ahmad, Habab Ali; Khan, Haleema; Shahzad, Muhammad; Haq, Zia ul; Harakeh, Steve; Yousafzai, Yasar Mehmood	2022	not Omicron period
Decreased Mortality From SARS-CoV-2 in Kidney Transplant Recipients Over the Course of the Pandemic	Al Azzi, Y.; Liriano-Ward, L. E.; Kapoor, S.; Ajaimy, M.; Akalin, E.; Pynadath, C. T.	2022	no full text

Title	Authors	Published Year	Reason for exclusion
Re-positive PCR of SARS-CoV-2 in health care persons during COVID-19 pandemic	Alebouyeh, M.; Aavani, P.; Abdulrahman, N. A.; Haleem, A. A.; Karimi, A.; Armin, S.; Fallah, F.; Amirali, A.; Sadr, S.; Ghandchi, G.; Abdollahi, N.; Ghanaie, R. M.; Tabatabaei, S. R.; Fahimzad, S. A.; Razmara, R.; Alzahrani, K. J.; Khanbabaee, G.; Vaghefi, S. S.; Imanzadeh, F.; Eshghi, P.; Azimi, L.	2021	not Omicron period
SARS-CoV-2 Reinfection Rate: A Systematic Review and Meta-analysis	Alimohamadi, Y.; Bahani, K.; Alimohammadi, K.; Sepandi, M.	2023	not Omicron period
Protective Effect of Previous SARS-CoV-2 Infection against Omicron BA.4 and BA.5 Subvariants	Altarawneh, H. N.; Chemaitelly, H.; Ayoub, H. H.; Hasan, M. R.; Coyle, P.; Yassine, H. M.; Al-Khatib, H. A.; Smatti, M. K.; Al-Kanaani, Z.; Al-Kuwari, E.; Jeremijenko, A.; Kaleeckal, A. H.; Latif, A. N.; Shaik, R. M.; Abdul-Rahim, H. F.; Nasrallah, G. K.; Al-Kuwari, M.; Butt, A. A.; Al-Romaihi, H. E.; Al-Thani, M. H.; Al-Khal, A.; Bertollini, R.; Tang, P.; Abu-Raddad, L. J.	2022	wrong outcomes
Active pharmacovigilance in patients immunized with the CoronaVac vaccine in the port of Cartagena group	Alviz-Amador, A.; Moreno-Babilonia, L.; Tulcan-Canchala, K. M.; Contreras-Puentes, N.; Gomez-Castillo, L. E.; Ramos-Clason, E.	2022	no full text
COVID REINFECTIONS SURGE DURING OMICRON WAVE	Anonymous,	2022	wrong study design
Clinical and laboratory outcomes of the solid cancer patients reinfected with SARS-CoV-2	Ãœnsal, Oktay; Yazici, Ozan; Ã–zdemir, Nuriye; Ã‡ubukÃ§u, Erdem; Ocak, Birol; Ãœner, Aytug; Ã–zet, Ahmet	2022	not Omicron period
Role of previous infection with SARS-CoV-2 in protecting against omicron reinfections and severe complications of COVID-19 compared to pre-omicron variants: a systematic review	Arabi, Maryam; Al-Najjar, Yousef; Sharma, Omna; Kamal, Ibtihal; Javed, Aimen; Gohil, Harsh S.; Paul, Pradipta; Al-Khalifa, Aljazi M.; Laws, Sa'ad; Zakaria, Dalia	2023	Wrong study design
COVID-19 Relapse, Reinfection Frequency, and Clinical Features of Cases	Aslaner, HÃ¼meyra; Aslaner, HacÄ± Ahmet; Savranlar, Yasemin; BenlÄ°, Ali Ramazan	2022	not Omicron period

Title	Authors	Published Year	Reason for exclusion
Susceptibility to reinfection with SARS-CoV-2 virus relative to existing antibody concentrations and T cell response	Atef, Shereen; Al Hosani, Farida; AbdelWareth, Laila; Al-Rifai, Rami H.; Abuyadek, Rowan; Jabari, Andrea; Ali, Raghib; Altrabulsi, Basel; Dunachie, Susanna; Alatoom, Adnan; Donnelly, James G.	2023	wrong outcomes
Evaluation of Newborns Diagnosed with COVID-19: A Single-Center Experience	Aydogan, Seda; Zenciroglu, Aysegul; Ã‡itli, Rumeysa; Dilli, Dilek; Ã–zdem, Suna	2022	not Omicron period
Covid-19 in Children with Chronic Kidney Disease; Does It Differ Much?	Baltu, D.; Kurt Sukur, E. D.; Ozturk, T. T.; Gulhan, B.; Ozaltin, F.; Duzova, A.; Topaloglu, R.	2022	no full text
Breakthrough infections in MPN-COVID vaccinated patients	Barbui, T.; Carobbio, A.; Ghirardi, A.; Iurlo, A.; De Stefano, V.; Sobas, M. A.; Rumi, E.; Elli, E. M.; Lunghi, F.; Gasior Kabat, M.; Cuevas, B.; Guglielmelli, P.; Bonifacio, M.; Marchetti, M.; Alvarez-Larran, A.; Fox, L.; Bellini, M.; Daffini, R.; Benevolo, G.; Carreno-Tarragona, G.; Patriarca, A.; Al-Ali, H. K.; Andrade-Campos, M. M. M.; Palandri, F.; Harrison, C.; Foncillas, M. A.; Osorio, S.; Koschmieder, S.; Magro Mazo, E.; Kiladjian, J. J.; Bolanos Calderon, E.; Heidel, F. H.; Quiroz Cervantes, K.; Griesshammer, M.; Garcia-Gutierrez, V.; Sanchez, A. M.; Hernandez-Boluda, J. C.; Lopez Abadia, E.; Carli, G.; Sagues Serrano, M.; Kusec, R.; Xicoy Cirici, B.; Guenova, M.; Navas Elorza, B.; Angona, A.; Cichocka, E.; Kulikowska de Nalecz, A.; Cattaneo, D.; Bucelli, C.; Betti, S.; Borsani, O.; Cavalca, F.; Carbonell, S.; Curto-Garcia, N.; Benajiba, L.; Rambaldi, A.; Vannucchi, A. M.	2022	unclear/ wrong reinfection definition
Risk and severity of SARS-CoV-2 reinfection among patients with multiple sclerosis vs. the general population: a population-based study	Barzegar, M.; Manteghinejad, A.; Bagherieh, S.; Sindarreh, S.; Mirmosayyeb, O.; Javanmard, S. H.; Shaygannejad, V.; Nasirian, M.	2022	not Omicron period
Clinical Characteristics and Outcomes of Kidney Transplant Recipients With SARS-CoV-2 Reinfections	Basic-Jukic, N.; Arnol, M.; Maksimovic, B.; Aleckovic-Halilovic, M.; Racki, S.; Barbic, J.; Babovic, B.; Juric, I.; Furic-Cunko, V.; Katalinic, L.; Radulovic, G.; Mihaljevic, D.; Jelakovic, B.; Kastelan, Z.	2022	not Omicron period

Title	Authors	Published Year	Reason for exclusion
Impact of the Omicron variant on SARS-CoV-2 reinfections in France, March 2021 to February 2022	Bastard, Jonathan; Taisne, Benjamin; Figoni, Julie; Mailles, Alexandra; Durand, Julien; Fayad, Myriam; Josset, Laurence; Maisa, Anna; van der Werf, Sylvie; Parent du ChÃ¢telet, Isabelle; Bernard-Stoecklin, Sibylle	2022	not Omicron period
Burden of COVID-19 disease and vaccine coverages in Apulian splenectomized patients: A retrospective observational study	Bianchi, F. P.; Stefanizzi, P.; Rizzi, D.; Signorile, N.; Cuscianna, E.; Daleno, A.; Migliore, G.; Tafuri, S.	2023	not Omicron period
Determinants of reinfection with SARS-CoV-2 Omicron variant	Bisharat, N.; Campisi-Pinto, S.	2023	wrong outcomes
COVID-19 vaccine effectiveness against severe disease from SARS-CoV-2 Omicron BA.1 and BA.2 subvariants - surveillance results from southern Sweden, December 2021 to March 2022	Bjork, J.; Bonander, C.; Moghaddassi, M.; Rasmussen, M.; Malmqvist, U.; Inghammar, M.; Kahn, F.	2022	wrong outcomes
Surveillance of COVID-19 vaccine effectiveness: a real-time case-control study in southern Sweden	Bjork, J.; Bonander, C.; Moghaddassi, M.; Rasmussen, M.; Malmqvist, U.; Kahn, F.; Inghammar, M.	2022	not Omicron period
Acute and postacute sequelae associated with SARS-CoV-2 reinfection	Bowe, B.; Xie, Y.; Al-Aly, Z.	2022	not Omicron period
COVID-19 vaccine effectiveness against symptomatic infection and hospitalisation in Belgium, July 2021 to May 2022	Braeye, T.; van Loenhout, J. A. F.; Brondeel, R.; Stouten, V.; Hubin, P.; Billuart, M.; Chung, P. Y. J.; Vandromme, M.; Wyndham-Thomas, C.; Blot, K.; Catteau, L.	2023	wrong outcomes
Early Omicron infection is associated with increased reinfection risk in older adults in long-term care and retirement facilities	Breznik, J. A.; Rahim, A.; Zhang, A.; Ang, J.; Stacey, H. D.; Bhakta, H.; Clare, R.; Liu, L. M.; Kennedy, A.; Hagerman, M.; Kajaks, T.; Miller, M. S.; Nazy, I.; Bramson, J. L.; Costa, A. P.; Bowdish, D. M. E.	2023	unclear/ wrong reinfection definition

Title	Authors	Published Year	Reason for exclusion
Protection from Omicron Infection in Residents of Nursing and Retirement Homes in Ontario, Canada	Breznik, Jessica A.; Rahim, Ahmad; Kajaks, Tara; Hagerman, Megan; Bilaver, Lucas; Colwill, Karen; Dayam, Roaya M.; Gingras, Anne-Claude; Verschoor, Chris P.; McElhaney, Janet E.; Bramson, Jonathan L.; Bowdish, Dawn M. E.; Costa, Andrew P.	2023	unclear/ wrong reinfection definition
Analysis of well-annotated next-generation sequencing data reveals increasing cases of SARS-CoV-2 reinfection with Omicron	Burkholz, Scott; Rubsamen, Michael; Blankenberg, Luke; Carback, Richard T.; Mochly-Rosen, Daria; Harris, Paul E.	2023	wrong study design
Protection against omicron (B.1.1.529) BA.2 reinfection conferred by primary omicron BA.1 or pre-omicron SARS-CoV-2 infection among health-care workers with and without mRNA vaccination: a test-negative case-control study	Carazo, Sara; Skowronski, Danuta M.; Brisson, Marc; Barkati, Sapha; Sauvageau, Chantal; Brousseau, Nicholas; Gilca, Rodica; Fafard, Judith; Talbot, Denis; Ouakki, Manale; Gilca, Vladimir; Carignan, Alex; Deceuninck, GeneviÃ¨ve; De Wals, Philippe; De Serres, Gaston	2022	unclear/ wrong reinfection definition
Estimated Protection of Prior SARS-CoV-2 Infection Against Reinfection With the Omicron Variant Among Messenger RNA-Vaccinated and Nonvaccinated Individuals in Quebec, Canada	Carazo, Sara; Skowronski, Danuta M.; Brisson, Marc; Sauvageau, Chantal; Brousseau, Nicholas; Gilca, Rodica; Ouakki, Manale; Barkati, Sapha; Fafard, Judith; Talbot, Denis; Gilca, Vladimir; Deceuninck, GeneviÃ¨ve; Garenc, Christophe; Carignan, Alex; De Wals, Philippe; De Serres, Gaston	2022	wrong outcomes
Risk of SARS-CoV-2 Reinfections in a Prospective Inception Cohort Study: Impact of COVID-19 Vaccination	Casado, JosÃ© L.; Haemmerle, Johannes; Vizcarra, Pilar; Ramirez-Alonso, Gema; Salazar-Tosco, Andrea; Romero-Hernandez, Beatriz; Blasco, Magdalena; Rodriguez-Dominguez, Mario; Mirabella, Itria G.; Vallejo, Alejandro; Fernandez-Escribano, Marina	2022	not Omicron period

Title	Authors	Published Year	Reason for exclusion
Primary SARS-CoV-2 Infections, Re-infections and Vaccine Effectiveness during the Omicron Transmission Period in Healthcare Workers of Trieste and Gorizia (Northeast Italy), 1 December 2021-31 May 2022	Cegolon, Luca; Negro, Corrado; Mastrangelo, Giuseppe; Filon, Francesca Larese	2022	duplicate data (other publication included d/t longer study period and larger population size)
SARS CoV-2 Re infection after natural infection compared with previously sero-negative: Descriptive longitudinal study	Chaklader, B.; Srivastava, K.; Rathod, H.; Jadhav, S.; Bhawalkar, J.; Thakur, K.; Verma, P.	2022	not Omicron period
Long-term impact of COVID-19 among maintenance haemodialysis patients	Chawki, S.; Buchard, A.; Sakhi, H.; Dardim, K.; El Sakhawi, K.; Chawki, M.; Boulanger, H.; Kofman, T.; Dahmane, D.; Rieu, P.; Attaf, D.; Ahriz-Saksi, S.; Masoumi, A.; Diddaoui, A. Z.; Fromentin, L.; Michaut, P.; Nebbad, R.; Desassis, J. F.; Nicolet, L.; Sohier-Attias, J.; Besson, F.; Boula, R.; Hafi, A.; Ghazali, A.; Lamriben, L.; Arezki, A.; Dupuis, E.; Rifard, M. K.; Joly, D.; Attias, P.; El Karoui, K.	2022	not Omicron period
EFFECT OF BNT162B2 ANTIGEN DOSAGE ON PROTECTION AGAINST SARS-CoV-2 OMICRON INFECTION	Chemaitelly, H.; Ayoub, H. H.; Abu-Raddad, L. J.	2023	wrong outcomes
Protection of Omicron sub-lineage infection against reinfection with another Omicron sub-lineage	Chemaitelly, H.; Ayoub, H. H.; Coyle, P.; Tang, P.; Yassine, H. M.; Al-Khatib, H. A.; Smatti, M. K.; Hasan, M. R.; Al-Kanaani, Z.; Al-Kuwari, E.; Jeremijenko, A.; Kaleeckal, A. H.; Latif, A. N.; Shaik, R. M.; Abdul-Rahim, H. F.; Nasrallah, G. K.; Al-Kuwari, M. G.; Butt, A. A.; Al-Romaihi, H. E.; Al-Thani, M. H.; Al-Khal, A.; Bertollini, R.; Abu-Raddad, L. J.	2022	unclear/ wrong reinfection definition

Title	Authors	Published Year	Reason for exclusion
Protection from previous natural infection compared with mRNA vaccination against SARS-CoV-2 infection and severe COVID-19 in Qatar: a retrospective cohort study	Chemaitelly, Hiam; Ayoub, Houssein H.; AlMukdad, Sawsan; Coyle, Peter; Tang, Patrick; Yassine, Hadi M.; Al-Khatib, Hebah A.; Smatti, Maria K.; Hasan, Mohammad R.; Al-Kanaani, Zaina; Al-Kuwari, Einas; Jeremijenko, Andrew; Kaleeckal, Anvar Hassan; Latif, Ali Nizar; Shaik, Riyazuddin Mohammad; Abdul-Rahim, Hanan F.; Nasrallah, Gheyath K.; Al-Kuwari, Mohamed Ghaith; Butt, Adeel A.; Al-Romaihi, Hamad Eid; Al-Thani, Mohamed H.; Al-Khal, Abdullatif; Bertollini, Roberto; Abu-Raddad, Laith J.	2022	not Omicron period
Protection against Reinfection with the Omicron BA.2.75 Subvariant	Chemaitelly, Hiam; Tang, Patrick; Coyle, Peter; Yassine, Hadi M.; Al-Khatib, Hebah A.; Smatti, Maria K.; Hasan, Mohammad R.; Ayoub, Houssein H.; Altarawneh, Heba N.; Al-Kanaani, Zaina; Al-Kuwari, Einas; Jeremijenko, Andrew; Kaleeckal, Anvar H.; Latif, Ali N.; Shaik, Riyazuddin M.; Abdul-Rahim, Hanan F.; Nasrallah, Gheyath K.; Al-Kuwari, Mohamed G.; Butt, Adeel A.; Al-Romaihi, Hamad E.; Al-Thani, Mohamed H.; Al-Khal, Abdullatif; Bertollini, Roberto; Abu-Raddad, Laith J.	2023	wrong outcomes
Epidemiological characteristics of the three waves of COVID-19 epidemic in Taiwan during April 2022 to March 2023	Chen, Yi-Hsuan; Cheuh, Yu-Neng; Chen, Chiu-Mei; Kuo, Hung-Wei	2023	unclear/ wrong reinfection definition
Protection against Omicron from Vaccination and Previous Infection in a Prison System	Chin, E. T.; Leidner, D.; Lamson, L.; Lucas, K.; Studdert, D. M.; Goldhaber-Fiebert, J. D.; Andrews, J. R.; Salomon, J. A.	2022	wrong outcomes
Coronavirus disease 2019 (COVID-19) reinfection rates in Malawi: a possible tool to guide vaccine prioritisation and immunisation policies	Chisale, M. R. O.; Sinyiza, F. W.; Kaseka, P. U.; Chimbatata, C. S.; Mbakaya, B. C.; Wu, TsungShu; Nyambalo, B. W.; Chauma-Mwale, A.; Chilima, B.; Yu, KwongLeung; Kayira, A. B.	2023	not Omicron period
[Epidemiological characteristics of reinfection of 2019-nCoV and influencing factors in Ningbo]	Chu, Y. R.; Chen, Y.; Lei, S.; Zhang, Y. W.; Yi, B.; Ma, J. M.; Yan, K. D.; Wang, Y.; Li, B. J.; Lyu, M. Q.; Xu, G. Z.; Zhang, D. L.	2023	no full text

Title	Authors	Published Year	Reason for exclusion
Three Outbreaks of COVID-19 in a Single Nursing Home over Two Years of the SARS-CoV-2 Pandemic	Cokic, V.; Popovska, Z.; Lijeskic, O.; Sabic, L.; Djurkovic-Djakovic, O.	2023	unclear/ wrong reinfection definition
Risk factors for SARS-CoV-2 infection and epidemiological profile of Brazilian anesthesiologists during the COVID-19 pandemic: cross-sectional study	Costa, Luiz Guilherme Villares da; Monteiro, Frederico de Lima Jacy; Souza, JÃºlia Koerich de; Queiroz, VerÃ´nica Neves Fialho; Papa, FÃ¡bio de Vasconcelos	2022	not Omicron period
Characteristics and Outcomes of Patients with SARS-CoV-2 Reinfections Requiring Treatment in a COVID-19 Ambulatory Treatment Program	Cowman, K.; Golia, A.; Guo, Y.; McSweeney, T. D.; Chang, M. H.; Bao, H.; Nori, P.	2022	no full text
From Acute Phase to Long COVID: A Cross-Sectional Study of the Epidemiological Profile and Clinical Evaluation of SARS-CoV-2 Infection in Employees at a Pediatric Hospital	da Silva, M. R. T.; Costa, A. P.; da Luz, A. A.; Pelaio, C. H.; Cruz, F. B.; Steil, G. F.; Giamberardino, H. I. G.; Prando, C.	2023	not Omicron period
CaracterÃsticas epidemiolÃ³gicas de las reinfecciones por COVID-19 en los trabajadores sanitarios	DalÃ, A. P.; RodrÃguez, R. G.; RodrÃguez, M. P.; VÃ¡zquez, I. D.; FariÃ±as, F. F.; Rivas, M. C.	2022	no full text
Is HCV elimination among persons living with HIV feasible? Data from the NoCo study in the setting of the ICONA cohort	d'Arminio Monforte, A.; Tavelli, A.; Rossotti, R.; Gagliardini, R.; Saracino, A.; Lo Caputo, S.; Sala, M.; Quiros-Roldan, E.; Mussini, C.; Girardi, E.; Cozzi-Lepri, A.; Antinori, A.; Puoti, M.	2023	wrong outcomes

Title	Authors	Published Year	Reason for exclusion
Outcomes of laboratory-confirmed SARS-CoV-2 infection during resurgence driven by Omicron lineages BA.4 and BA.5 compared with previous waves in the Western Cape Province, South Africa	Davies, M. A.; Morden, E.; Rosseau, P.; Arendse, J.; Bam, J. L.; Boloko, L.; Cloete, K.; Cohen, C.; Chetty, N.; Dane, P.; Heekes, A.; Hsiao, N. Y.; Hunter, M.; Hussey, H.; Jacobs, T.; Jassat, W.; Kariem, S.; Kassanjee, R.; Laenen, I.; Roux, S. L.; Lessells, R.; Mahomed, H.; Maughan, D.; Meintjes, G.; Mendelson, M.; Mnguni, A.; Moodley, M.; Murie, K.; Naude, J.; Ntusi, N. A. B.; Paleker, M.; Parker, A.; Pienaar, D.; Preiser, W.; Prozesky, H.; Raubenheimer, P.; Rossouw, L.; Schrueder, N.; Smith, B.; Smith, M.; Solomon, W.; Symons, G.; Taljaard, J.; Wasserman, S.; Wilkinson, R. J.; Wolmarans, M.; Wolter, N.; Boulle, A.	2022	wrong outcomes
Evaluation of persistent COVID and SARS-CoV-2 reinfection in a cohort of patients on the island of Gran Canaria, Spain	de Arriba FernÃ¡ndez, A.; Bilbao, J. L. A.; FrancÃ©s, A. E.; Mora, A. C.; PÃ©rez, Ã G.; Barreiros, M. Ã D.	2023	not Omicron period
EvaluaciÃ³n del COVID-19 persistente y la reinfecciÃ³n por SARS-CoV-2 en una cohorte de pacientes en la isla de Gran Canaria, EspaÃ±a Evaluation of persistent COVID and SARS-CoV-2 reinfection in a cohort of patients on the island of Gran Canaria, Spain	de Arriba FernÃ¡ndez, Alejandro Luis Alonso Bilbao JosÃ© FrancÃ©s Alberto EspiÃ±eira Mora Antonio Cabeza PÃ©rez Ãngela GutiÃ©rrez Barreiros Miguel Ãngel DÃaz	2023	not Omicron period
Risk of SARS-CoV-2 reinfection: a systematic review and meta-analysis	Deng, Luojia; Li, Peiqi; Zhang, Xuezhixing; Jiang, Qianxue; Turner, DeAnne; Zhou, Chao; Gao, Yanxiao; Qian, Frank; Zhang, Ci; Lu, Hui; Zou, Huachun; Vermund, Sten H.; Qian, Han-Zhu	2022	not Omicron period
Severity and Outcomes of SARS-CoV-2 Reinfection Compared with Primary Infection: A Systematic Review and Meta-Analysis	Deng, J.; Ma, Y.; Liu, Q.; Du, M.; Liu, M.; Liu, J.	2023	Wrong study design

Title	Authors	Published Year	Reason for exclusion
The mystery of COVID-19 reinfections: A global systematic review and meta-analysis	Dhillon, R. A.; Qamar, M. A.; Gilani, J. A.; Irfan, O.; Waqar, U.; Sajid, M. I.; Mahmood, S. F.	2021	wrong study design
Waning protection after vaccination and prior infection against COVID-19-related mortality over 18 months	Dietler, D.; Kahn, F.; Inghammar, M.; Bjork, J.	2023	wrong outcomes
Risk of disease relapse following COVID-19 vaccination in patients with AQP4-IgG-positive NMOSD and MOGAD	Dinoto, A.; Sechi, E.; Ferrari, S.; Gajofatto, A.; Orlandi, R.; Solla, P.; Maccabeo, A.; Maniscalco, G. T.; Andreone, V.; Sartori, A.; Manganotti, P.; Rasia, S.; Capra, R.; Mancinelli, C. R.; Mariotto, S.	2022	not Omicron period
Time dependent decline of neutralizing antibody titers in COVID-19 patients from Pune, India and evidence of reinfection	Doke, P.; Gothankar, J. S.; Doke, P. P.; Kulkarni, M. M.; Khalate, K. K.; Shrivastava, S.; Patil, J. R.; Arankalle, V. A.	2022	not Omicron period
Association of Spike-Specific T Cells With Relative Protection From Subsequent SARS-CoV-2 Omicron Infection in Young Children	Dowell, Alexander C.; Ireland, Georgina; Zuo, Jianmin; Moss, Paul; Ladhani, Shamez	2022	unclear/ wrong reinfection definition
Analysis of recurrent positive COVID-19 patients in a hospital	Durmus, Ensar Guneysu Fatih	2021	not Omicron period
Daily Rapid Antigen Exit Testing to Tailor University COVID-19 Isolation Policy	Earnest, R.; Chen, C.; Chaguza, C.; Hahn, A. M.; Grubaugh, N. D.; Wilson, M. S.; Cairns, A.; Cooksey, A.; Pensiero, A.; Mosha, A.; Pivirotto, C.; Traub, C.; Velazquez, G.; Lee, H.; Larioza, J.; Neal, J.; Bourgeois, J.; Donnelly, K.; Otterson, K.; Carbone, K.; Smith, K.; Gillingham, L.; Greenberg, L.; Di Gangi Ehrenfels, L.; Adileh, M.; Toupou, M.; Lubich, N.; Yuen, N.; Davies, N.; Brown, N.; Khalid, N.; Dumont, O.; Umeugo, O.; Monteagudo, O.; Hill, P.; O'Leary, Q.; Banks, R.; Rousseau, R.; Duplantis, S.; Khalid, S.; Cesar, S.; Doran, S.; Charney, S.; Wilkins, S.; Martinez, S.; Volpe, S.; Garcia, V. M.; Cappuccia, V.	2022	wrong outcomes

Title	Authors	Published Year	Reason for exclusion
Evaluation of possible COVID-19 reinfection in children: A multicenter clinical study: COVID-19 reinfection in children	Erbas, I. C.; Keles, Y. E.; Erdeniz, E. H.; Yilmaz, A. T.; Yesil, E.; Cakici, O.; Akca, M.; Ulu, N. K.; Dinc, F.; Ciftdogan, D. Y.; Oncel, S.; Kuyucu, N.; Tapisiz, A.; Belet, N.	2023	not Omicron period
Prior SARS-COV-2 infection, vaccination, COVID-19 hospital admission and mortality amongst nursing home residents: comment...Kleebayoon A, Wiwanitkit V. Aging Clinical & Experimental Research. 2023;35(9):1977-1977. 1p	EspaÃ±a, Pedro P.; Bilbao-GonzÃ¡lez, Amaia; Quintana, Jose M.	2023	wrong study design
Reincidence Epidemiological Analysis for Positive Covid-19 Cases in Mexico	Fajardo-Montiel, A.; Castellanos-Tadeo, C. A.; Godinez, H. H. U.; Ramirez-Sanchez, H. U.; Garcia-Guadalupe, M. E.; Garcia-Concepcion, F. O.	2022	no full text
COVID-19 vaccines reduce the risk of SARS-CoV-2 reinfection and hospitalization: Meta-analysis	Flacco, M. E.; Acuti Martellucci, C.; Baccolini, V.; De Vito, C.; Renzi, E.; Villari, P.; Manzoli, L.	2022	not Omicron period
Risk of SARS-CoV-2 reinfection 18 months after first infection: population-level observational study : Maria Elena Flacco	Flacco, M. E.; Acuti Martellucci, C.; Soldato, G.; Di Martino, G.; Carota, R.; Caponetti, A.; Manzoli, L.	2022	no full text
Risk of reinfection and disease after SARS-CoV-2 primary infection: Meta-analysis	Flacco, M. E.; Acuti Martellucci, C.; Baccolini, V.; De Vito, C.; Renzi, E.; Villari, P.; Manzoli, L.	2022	Wrong study design

Title	Authors	Published Year	Reason for exclusion
Risk and symptoms of COVID-19 in health professionals according to baseline immune status and booster vaccination during the Delta and Omicron waves in Switzerland-A multicentre cohort study	Flury, B. B.; Gusewell, S.; Egger, T.; Leal, O.; Brucher, A.; Lemmenmeier, E.; Meier Kleeb, D.; Moller, J. C.; Rieder, P.; Rutti, M.; Schmid, H. R.; Stocker, R.; Vuichard- Gysin, D.; Wiggli, B.; Besold, U.; McGeer, A.; Risch, L.; Friedl, A.; Schlegel, M.; Kuster, S. P.; Kahlert, C. R.; Kohler, P.	2022	not Omicron period
Prevalence and impact factors of recurrent positive SARS-CoV-2 detection in 599 hospitalized COVID-19 patients	Gao, C.; Zhu, L.; Jin, C. C.; Tong, Y. X.; Xiao, A. T.; Zhang, S.	2021	not Omicron period
Repeated respiratory viral infections in children	Gao, L.; Shen, K.	2021	wrong study design
Evidence of early community transmission of Omicron (B1.1.529) in Delhi- A city with very high seropositivity and past-exposure	Garg, R.; Gautam, P.; Suroliya, V.; Agarwal, R.; Bhugra, A.; Kaur, U. S.; Das, S.; Bihari, C.; Agarwal, A.; Sarin, S. K.; Gupta, E.	2022	wrong outcomes
Real-life data on monoclonal antibodies and antiviral drugs in Italian inborn errors of immunity patients during COVID-19 pandemic	Garzi, G.; Cinetto, F.; Firinu, D.; Di Napoli, G.; Lagnese, G.; Punziano, A.; Bez, P.; Cinicola, B. L.; Costanzo, G.; Scarpa, R.; Pulvirenti, F.; Rattazzi, M.; Spadaro, G.; Quinti, I.; Milito, C.	2022	not Omicron period
The Incidence of SARS-CoV-2 Reinfection in Persons With Naturally Acquired Immunity With and Without Subsequent Receipt of a Single Dose of BNT162b2 Vaccine : A Retrospective Cohort Study	Gazit, Sivan; Shlezinger, Roei; Perez, Galit; Lotan, Roni; Peretz, Asaf; Ben-Tov, Amir; Herzel, Esma; Alapi, Hillel; Cohen, Dani; Muhsen, Khitam; Chodick, Gabriel; Patalon, Tal	2022	not Omicron period
Sarscov-2 virus reinfection in a healthcare worker	Giraldo-Ospina, C. E.; Giraldo-Lopez, K.	2021	wrong study design

Title	Authors	Published Year	Reason for exclusion
Sars-Cov-2 Infection and Depressive Symptoms among Frail Individuals Living in Long-Term Care Facility	Gouin, J. P.; Cruz-Santiago, D.; Canac-Marquis, M.; Vinh, D.	2023	no full text
Recurrent SARS-CoV-2 RNA Detection after COVID-19 Illness Onset during Pregnancy	Griffin, Isabel; Woodworth, Kate R.; Galang, Romeo R.; Burkel, Veronica K.; Neelam, Varsha; Siebman, Samantha; Barton, Jerusha; Manning, Susan E.; Aveni, Kathryn; Longcore, Nicole D.; Harvey, Elizabeth M.; Ngo, Van; Mbotha, Deborah; Chicchelly, Sarah; Lush, Mamie; Eckert, Valorie; Dzimira, Paula; Sokale, Ayomide; Valencia-Prado, Miguel; Azziz-Baumgartner, Eduardo; MacNeil, Adam; Gilboa, Suzanne M.; Tong, Van T.	2022	not Omicron period
Occurrence of SARS-CoV-2 reinfections at regular intervals in Ecuador	Guevara, Rommel; Prado-Vivar, BelÃ©n; MÃ¡rquez, Sully; MuÃ±oz, Erika B.; Carvajal, Mateo; Guadalupe, Juan JosÃ©; Becerra-Wong, MÃ³nica; ProaÃ±o, Stefanie; Bayas-Rea, Rosa; Coloma, Josefina; Grunauer, Michelle; Trueba, Gabriel; Rojas-Silva, Patricio; BarragÃ¡n, VerÃ³nica; CÃ¡rdenas, PaÃºl	2022	unclear/ wrong reinfection definition
Morbidity and Mortality of Sars-Cov-2 Positive Hospitalised Hemodialysis Patients in Case of Centralized Organizational Structure in Healthcare	Hazoyan, A.	2023	no full text
Risk for Reinfection after SARS-CoV-2: A Living, Rapid Review for American College of Physicians Practice Points on the Role of the Antibody Response in Conferring Immunity following SARS-CoV-2 Infection	Helfand, M.; Fiordalisi, C.; Wiedrick, J.; Ramsey, K. L.; Armstrong, C.; Gean, E.; Winchell, K.; Arkhipova-Jenkins, I.	2022	not Omicron period

Title	Authors	Published Year	Reason for exclusion
Risk of Reinfection From SARS-CoV-2 â€“ An Update of an Antibody Response Following SARS-CoV-2 Infection and Implications for Immunity: A Living Rapid Review	Helfand, Mark; Fiordalisi, Celia; Wiedrick, Jack; Ramsey, Katrina L.; Armstrong, Charlotte; Gean, Emily; Winchell, Kara; Arkhipova-Jenkins, Irina	2022	wrong study design
Demographic and Clinical Characteristics of COVID-19 Reinfection	Hoard, R.; Coate, E.; Macias, E. A.; Jackson, L. B.; Barsoumian, A. E.; Okulicz, J.; Markelz, E.	2022	no full text
Emergent Omicron BR.2.1 sublineage of SARS-CoV-2 in New South Wales, Australia: a subvariant with high fitness but without increased disease severity	Howard-Jones, Annaleise R.; Arnott, Alicia; Draper, Jenny; Gall, Mailie; Ellis, Sally; Marris, Kelsi; Selvey, Christine; Basile, Kerri; Dwyer, Dominic E.; Sintchenko, Vitali; Kok, Jen	2023	unclear/ wrong reinfection definition
Characteristics of patients with SARS-COV-2 PCR re-positivity after recovering from COVID-19	Hu, Cheng-Yi; Lei, Yi; Tang, Yu-Wen; Cui, Wen-Shuai; Wu, Pei-Lian; Li, Yan-Fang; Zhou, Yan; Li, Xin-Yan; Cui, Hao; Xiao, Lu-Shan; Zhao, Zhu-Xiang	2023	not Omicron period
Reinfection by SARS CoV2 in Valle Del Cauca, Colombia: A Descriptive Retrospective Study	Hurtado, Isabel Cristina; Hurtado, Juan SebastiÃ¡n; Valencia, Sandra Lizeth; PinzÃ³n, Elisa MarÃa; GuzmÃ¡n, Ana RociÃ³; Lesmes, MarÃa Cristina	2022	not Omicron period
Clinical severity according to the primary infection variant in patients with suspected SARS-CoV-2 reinfection in Korea	Hwang, Myung-Jae; Hwang, Insob; Park, Chungmin; Park, Hanul; Son, Taejong; Kim, Jong-Hun	2023	wrong outcomes
SARS-COV-2 infection in workers of health: epidemiological risk analisis in a highly complex institution Bogota, Colombia, 2020. [Spanish]	Ibanez-Pinilla, M.; Murcia, L.; Briceno, L.; Trillos, C. E.; Ramirez, C.; Daza, J.; Ballesteros, I.; Sanchez, J.; Rodriguez, G.	2022	not Omicron period

Title	Authors	Published Year	Reason for exclusion
Estimated Effectiveness of Prior SARS-CoV-2 BA.1 or BA.2 Infection and Booster Vaccination Against Omicron BA.5 Subvariant Infection	Jang, Eun Jung; Choe, Young June; Kim, Ryu Kyung; Lee, Sangwon; Park, Seon Kyeong; Park, Young-Joon	2023	wrong outcomes
Characteristics of Chemosensory Perception in Long COVID and COVID Reinfection	Jaramillo, Mikki; Thyvalikakath, Thankam P.; Eckert, George; Srinivasan, Mythily	2023	unclear/ wrong reinfection definition
SARS-CoV-2 Infections and Mortality in Dialysis Patients: A Multicenter Prospective Trial	Kamalanabhaiah, S. R.; Ostermaier, C.; Dahmen, L. L.; Haas, C. S.; Hoyer, J.; Keller, C.	2022	no full text
Reinfection rate and protection effectiveness from past infection among healthcare workers in public tertiary hospitals in Malaysia: A prospective cohort study	Karina, K.; Ven, L. J.; Lan, Y. S.; How, Y. C.; Kumar, C. A.; Adiratna, M. R.; Eliza, M. N.; Aisyah, A. R. N.; Gokilavanan, V.; Sevalingam, R. K.; Peariasamy, K. M.	2022	no full text
Outcome of SARS-CoV-2 infection among patients with common variable immunodeficiency and a matched control group: A Danish nationwide cohort study	Katzenstein, T. L.; Rasmussen, L. D.; Drabe, C. H.; Larsen, C. S.; Hansen, A. B. E.; Staerkind, M.; Knudsen, L. S.; Hansen, C. H.; Obel, N.	2022	wrong outcomes
Outcome of SARS-CoV-2 infection among patients with common variable immunodeficiency and a matched control group: A Danish nationwide cohort study	Katzenstein, Terese L.; Rasmussen, Line D.; Drabe, Camilla Helberg; Larsen, Carsten Schade; Hansen, Ann-Brit Eg; StÃ¦rkind, Mette; Knudsen, Lene Surland; Hansen, Christian Holm; Obel, Niels	2022	not Omicron period

Title	Authors	Published Year	Reason for exclusion
Vaccine escape, increased breakthrough and reinfection in infliximab-treated patients with IBD during the Omicron wave of the SARS-CoV-2 pandemic	Kennedy, N. A.; Janjua, M.; Chanchlani, N.; Lin, S.; Bewshea, C.; Nice, R.; McDonald, T. J.; Auckland, C.; Harries, L. W.; Davies, M.; Michell, S.; Kok, K. B.; Lamb, C. A.; Smith, P. J.; Hart, A. L.; Pollok, R. C.; Lees, C. W.; Boyton, R. J.; Altmann, D. M.; Sebastian, S.; Powell, N.; Goodhand, J. R.; Ahmad, T.	2023	not Omicron period
COVID-19 in patients with chronic lymphocytic leukemia: a Moscow observational study	Kochneva, O. L.; Kislova, M.; Zhelnova, E. I.; Petrenko, A. A.; Baryakh, E. A.; Yatskov, K. V.; Dmitrieva, E. A.; Misurina, E. N.; Nikitin, K. E.; Vasilieva, E. J.; Samsonova, I. V.; Ptushkin, V. V.; Baranova, A.; Nikitin, E. A.	2022	not Omicron period
Incidence of SARS-CoV-2 infection among previously infected or vaccinated employees	Kojima, N.; Roshani, A.; Brobeck, M.; Baca, A.; Klausner, J. D.	2022	not Omicron period
Evaluation of the Factors Associated with Reinfections towards SARS-CoV-2 Using a Case Control Design	La Torre, Giuseppe; Paglione, Gianluca; Barone, Lavinia Camilla; Cammalleri, Vittoria; Faticoni, Augusto; Marte, Mattia; Pocino, Roberta Noemi; Previte, Carlo Maria; Bongiovanni, Andrea; Colaprico, Corrado; Ricci, Eleonora; Imeshtari, Valentin; Manai, Maria Vittoria; Shaholli, David; Barletta, Vanessa India; Carluccio, Giovanna; Moretti, Luca; Vezza, Francesca; Volpicelli, Lorenzo; Massetti, Anna Paola; Cinti, Lilia; Roberto, Piergiorgio; Napoli, Anna; Antonelli, Guido; Mastroianni, Claudio Maria; Sernia, Sabina	2023	wrong outcomes
Epidemiological and Clinical Features of SARS-CoV-2 Variants Circulating between April-December 2021 in Italy	Lai, A.; Bergna, A.; Della Ventura, C.; Menzo, S.; Bruzzone, B.; Sagradi, F.; Ceccherini-Silberstein, F.; Weisz, A.; Clementi, N.; Brindicci, G.; Vicenti, I.; Sasset, L.; Caucci, S.; Corvaro, B.; Ippoliti, S.; Acciarri, C.; De Pace, V.; Lanfranchi, L.; Bellocchi, M. C.; Giurato, G.; Ferrarese, R.; Lagioia, A.; Francisci, D.; Colombo, M. L.; Lazzarin, S.; Ogliastro, M.; Cappelletti, M. R.; Iannetta, M.; Rizzo, F.; Torti, C.; Fumi, M.; D'Avenia, M.; Brusa, S.; Greco, F.; Menchise, A.; Letizia, V.; Vaccaro, E.; Santoro, C. R.; Fraccalvieri, C.; Testa, S.; Carioti, L.; Rocco, T.; Saracino, A.; Cattelan, A.; Clementi, M.; Sarmati, L.; Riva, A.; Galli, M.; Antinori, S.; Zehender, G.	2022	not Omicron period

Title	Authors	Published Year	Reason for exclusion
Serial infection with SARS-CoV-2 Omicron BA.1 and BA.2 following three-dose COVID-19 vaccination	Lapointe, H. R.; Mwimanzi, F.; Cheung, P. K.; Sang, Y.; Yaseen, F.; Kalikawe, R.; Datwani, S.; Waterworth, R.; Umviligihozo, G.; Ennis, S.; Young, L.; Dong, W.; Kirkby, D.; Burns, L.; Leung, V.; Holmes, D. T.; DeMarco, M. L.; Simons, J.; Matic, N.; Montaner, J. S. G.; Brumme, C. J.; Prystajecky, N.; Niikura, M.; Lowe, C. F.; Romney, M. G.; Brockman, M. A.; Brumme, Z. L.	2022	wrong study design
Case of Early Reinfection With Severe Acute Respiratory Syndrome Coronavirus 2 (SARS-CoV-2)	Larson, Derek; Brodniak, Sterling L.; Voegtly, Logan J.; Cer, Regina Z.; Glang, Lindsay A.; Malagon, Francisco J.; Long, Kyle A.; Potocki, Ronald; Smith, Darci R.; Lanteri, Charlotte; Burgess, Timothy; Bishop-Lilly, Kimberly A.	2021	wrong study design
Does pre-existing immunity determine the course of SARS-CoV-2 infection in health-care workers? Single-center experience	Laura, Luka; Dalmatin-Dragisic, Monika; Martinovic, Katarina; Tutis, Borka; Herceg, Ivana; Arapovic, Maja; Arapovic, Jurica	2022	wrong outcomes
Association of SARS-CoV-2 BA.4/BA.5 Omicron lineages with immune escape and clinical outcome	Lewnard, J. A.; Hong, V.; Kim, J. S.; Shaw, S. F.; Lewin, B.; Takhar, H.; Tartof, S. Y.	2023	wrong outcomes
Milder symptoms and shorter course in patients with re-positive COVID-19: A cohort of 180 patients from Northeast China	Li, Hongyan; Zhu, Mingqin; Zhang, Peng; Yan, Xingjian; Niu, Junqi; Wang, Zhenyu; Cao, Jie	2022	wrong outcomes
Analysis of Symptomology, Infectiveness, and Reinfections between Male and Female COVID-19 Patients: Evidence from Japanese Registry Data	Li, Meng-Hao Siddique Abu Bakkar Andalibi Ali Koizumi Naoru	2021	not Omicron period

Title	Authors	Published Year	Reason for exclusion
Clinical characteristics and risks of the convalescent COVID-19 patients with re-detectable positive RNA test: a 430 patients with Omicron infected cross-sectional survey in Tianjin, China	Li, Tianning; Han, Meng; Wang, Jingyu; Zhou, Chunlei; Mu, Hong	2022	wrong study design
SARS-CoV-2-specific T cell responses wane profoundly in convalescent individuals 10 months after primary infection	Li, Z.; Xiang, T.; Liang, B.; Deng, H.; Yang, X.; Wang, H.; Feng, X.; Zelinskyy, G.; Trilling, M.; Sutter, K.; Lu, M.; Dittmer, U.; Wang, B.; Yang, D.; Zheng, X.; Liu, J.	2023	wrong outcomes
Effects of Vaccination and Previous Infection on Omicron Infections in Children	Lin, D. Y.; Gu, Y.; Xu, Y.; Zeng, D.; Wheeler, B.; Young, H.; Sunny, S. K.; Moore, Z.	2022	wrong outcomes
Association of Primary and Booster Vaccination and Prior Infection With SARS-CoV-2 Infection and Severe COVID-19 Outcomes	Lin, Dan-Yu; Gu, Yu; Xu, Yangjianchen; Wheeler, Bradford; Young, Hayley; Sunny, Shadia Khan; Moore, Zack; Zeng, Donglin	2022	not Omicron period
A follow-up study on the recovery and reinfection of Omicron COVID-19 patients in Shanghai, China	Lin, M.; Cao, K.; Xu, F.; Wu, X.; Shen, Y.; Lu, S.; Kuang, Z.; Ding, H.; Yuan, S.; Shao, M.; Gu, G.; Xing, L.; Gu, T.; Chen, S.; Sun, J.; Zhu, J.; Zhang, X.; Yang, Y.; Zhao, G.; Huang, L.; Xu, J.; Song, Z.	2023	unclear/ wrong reinfection definition
Impact of COVID-19 pandemic in terms of incidence and lethality in nursing homes in Galicia (Spain)	Losada-Castillo, I.; Santiago-Perez, M. I.; Naveira-Barbeito, G.; Otero-Barros, M. T.; Perez-Martinez, O.; Zubizarreta-Alberdi, R.	2022	wrong language
Meta-analysis of SARS-CoV-2 reinfection rate in the world	Ma, Y. R.; Deng, J.; Liang, W.; Liu, M.; Liu, J.	2023	wrong study design
Clinical and Socio-Demographic Variables Associated With Long COVID-19: A Cross-Sectional Study	Mahmoodi, Zohreh; Bahrami, Giti; Shahrestanaki, Ehsan; Seddighi, Hamed; Ghavidel, Nooshin	2023	unclear/ wrong reinfection definition

Title	Authors	Published Year	Reason for exclusion
Evaluation of burden of the COVID-19 pandemic among the cystic fibrosis community in Brno, Czech Republic	Mala, M.; Homola, L.; Pokojova, E.; Stastna, N.; Holcikova, A.; Gracova, Z.	2022	no full text
Sars-Cov-2 Reinfection among Maintenance Dialysis Patients: Report of 471 Cases	Manley, H.; Hsu, C.; Li, N. C.; Shieu, M.; Harford, A.; Weiner, D.; Miskulin, D.; Johnson, D.; Lacson, E.	2023	no full text
Reinfection rates among patients previously infected by SARS-CoV-2: Systematic review and meta-analysis	Mao, Y.; Wang, W.; Ma, J.; Wu, S.; Sun, F.	2022	Wrong study design
SARS-CoV-2 reinfections during the first three major COVID-19 waves in Bulgaria	Marinov, Georgi K.; Mladenov, Mladen; Rangachev, Antoni; Alexiev, Ivailo	2022	not Omicron period
Risk and severity of SARS-CoV-2 reinfections during 2020-2022 in Vojvodina, Serbia: A population-level observational study	Medic, Snezana; Anastassopoulou, Cleo; Lozanov-Crvenkovic, Zagorka; Vukovic, Vladimir; Dragnic, Natasa; Petrovic, Vladimir; Ristic, Mioljub; Pustahija, Tatjana; Gojkovic, Zoran; Tsakris, Athanasios; Ioannidis, John P. A.	2022	wrong outcomes
Rise of COVID-19 Re-infections as Omicron Variant Prevailed: Implications on Monitoring the Course of the Pandemic	Mellou, K.; Gkolfinopoulou, K.; Tryfinopoulou, K.; Panagoulias, I.; Psallida, P.; Gerolymatos, G.; Tsiodras, S.; Panagiotakopoulos, G.; Paraskevis, D.; Zaoutis, T.	2022	no full text
Immediate reinfection with Omicron variant after clearance of a previous SARS-CoV-2 infection	Mencacci, Antonella; Gili, Alessio; Camilloni, Barbara; Bicchieraro, Giulia; Spaccapelo, Roberta; Bietta, Carla; Stracci, Fabrizio	2022	wrong outcomes
Risk of SARS-CoV-2 reinfections in children: a prospective national surveillance study between January, 2020, and July, 2021, in England	Mensah, A. A.; Campbell, H.; Stowe, J.; Seghezzo, G.; Simmons, R.; Lacy, J.; Bukasa, A.; O'Boyle, S.; Ramsay, M. E.; Brown, K.; Ladhani, S. N.	2022	not Omicron period

Title	Authors	Published Year	Reason for exclusion
COVID-19 Outcomes in Kidney Transplant Recipients in a German Transplant Center	Mikhailov, M.; Budde, K.; Halleck, F.; Eleftheriadis, G.; Naik, M. G.; Schrezenmeier, E.; Bachmann, F.; Choi, M.; Duettmann, W.; von Hoerschelmann, E.; Koch, N.; Liefeldt, L.; Lucht, C.; Straub-Hohenbleicher, H.; Waiser, J.; Weber, U.; Zukunft, B.; Osmanodja, B.	2023	unclear/ wrong reinfection definition
Impact of prior infection status on antibody response to the BNT162b2 mRNA COVID-19 vaccine in healthcare workers at a COVID-19 referral hospital in Milan, Italy	Milazzo, L.; Pezzati, L.; Oreni, L.; Kullmann, C.; Lai, A.; Gabrieli, A.; Bestetti, G.; Beschi, C.; Conti, F.; Ottomano, C.; Gervasoni, C.; Meroni, L.; Galli, M.; Antinori, S.; Ridolfo, A. L.	2021	not Omicron period
GLOBAL VIEW OF THE SARS-COV-2 PANDEMIC IN THE WORKING POPULATION OF THE NAVARRAN HEALTH SERVICE-OSASUNBIDEA. [Spanish]	Mollov, A.; Rubio, M.; Narvaez, C.; Jimenez, S.; Munoyerro, N.; Echeverria, A.; Asenjo, B.	2023	no full text
SARS-CoV-2 and Its Variants in Thrice-Infected Health Workers: A Case Series from an Italian University Hospital	Monaco, M. G. L.; Spiteri, G.; Caliskan, G.; Lotti, V.; Carta, A.; Gibellini, D.; Verlato, G.; Porru, S.	2022	wrong outcomes
Effectiveness of a Second Dose of an mRNA Vaccine Against Severe Acute Respiratory Syndrome Coronavirus 2 (SARS-CoV-2) Omicron Infection in Individuals Previously Infected by Other Variants	Monge, Susana; Rojas-Benedicto, AyelÃ©n; Olmedo, Carmen; MartÃn-Merino, Elisa; Mazagatos, Clara; Limia, Aurora; Sierra, MarÃa JosÃ©; Larrauri, Amparo; HernÃ¡n, Miguel A.; Ibercovid,	2023	unclear/ wrong reinfection definition
Protection of hybrid immunity against SARS-CoV-2 reinfection and severe COVID-19 during periods of Omicron variant predominance in Mexico	Montes-GonzÃ¡lez, JosÃ© Antonio; Zaragoza-JimÃ©nez, Christian Arturo; Antonio-Villa, Neftali Eduardo; FermÃn-MartÃnez, Carlos A.; RamÃrez-GarcÃa, Daniel; Vargas-VÃ¡zquez, Arsenio; GutiÃ©rrez-Vargas, Rosaura Idania; GarcÃa-RodrÃguez, Gabriel; LÃ³pez-Gatell, Hugo; ValdÃ©s-Ferrer, Sergio IvÃ¡n; Bello-Chavolla, Omar Yaxmehen	2023	wrong outcomes

Title	Authors	Published Year	Reason for exclusion
Reinfections from SARS-CoV-2: A Retrospective Study from the Gyncentrum Genetic Laboratory in Sosnowiec, Poland, April 2020 to July 2022	Morawiec, Emilia; Bednarska-Czerwinska, Anna; Pudelko, Adam; Zmarzly, Nikola; Rojczyk, Ewa; Madej, Krzysztof; Sobanski, Dawid; Staszkiewicz, Rafal; Ossowski, Piotr; Boron-Kaczmarska, Anna; Zapletal-Pudelko, Karolina; Boron, Dariusz; Grabarek, Beniamin Oskar	2023	not Omicron period
SARS-CoV-2 reinfections during the Delta and Omicron waves	Morris, C. P.; Eldesouki, R. E.; Fall, A.; Gaston, D. C.; Norton, J. M.; Gallagher, N. D.; Luo, C. H.; Abdullah, O.; Klein, E. Y.; Mostafa, H. H.	2022	wrong outcomes
Re-Infection with SARS-CoV-2 in Solid-Organ Transplant Recipients: Incidence Density and Convalescent Immunity Prior to Re-Infection	Morris, S.; Anjan, S.; Pallikkuth, S.; Frattaroli, P.; Courel, S.; Fernandez, A.; Natori, A.; Abbo, L.; Pahwa, S.; Guerra, G.; Natori, Y.	2022	no full text
Reinfection with SARS-CoV-2 in solid-organ transplant recipients: Incidence density and convalescent immunity prior to reinfection	Morris, Stephen; Anjan, Shweta; Pallikkuth, Suresh; Frattaroli, Paola; Courel, Steve; Fernandez, Anmary; Natori, Akina; Abbo, Lilian; Pahwa, Savita; Guerra, Giselle; Natori, Yoichiro	2022	not Omicron period
Symptomatic SARS-COV-2 reinfection: healthcare workers and immunosuppressed individuals at high risk	Murillo-Zamora, EfrÃ©n; Trujillo, XÃ³chitl; Huerta, Miguel; RÃos-Silva, MÃ³nica; Aguilar-Sollano, Felipe; Mendoza-Cano, Oliver	2021	not Omicron period
COVID-19 vaccines provide better protection against related pneumonia than previous symptomatic infection	Murillo-Zamora, EfrÃ©n; Trujillo, XÃ³chitl; Huerta, Miguel; RÃos-Silva, MÃ³nica; GuzmÃ¡n-Esquivel, JosÃ©; Benites-GodÃnez, VerÃ³nica; Ochoa-Castro, MarÃa Regina; GuzmÃ¡n-SolÃ³rzano, JosÃ© Alejandro; Mendoza-Cano, Oliver	2022	not Omicron period
High rate of reinfection with the SARS-CoV-2 Omicron variant	Nguyen, N. N.; Houhamdi, L.; Hoang, V. T.; Stoupan, D.; Fournier, P. E.; Raoult, D.; Colson, P.; Gautret, P.	2022	no full text
Infection and reinfection with SARS-CoV-2 in cancer patients: A cohort study	Nitipir, C.; Parosanu, A. I.; Olaru, M.; Popa, A. M.; Pirlog, C.; Iaciu, C.; Vrabie, R.; Stanciu, M. I.; Oprescu-Macovei, A.; Bumbacea, D.; Negrei, C.; Orlov-Slavu, C.	2022	not Omicron period

Title	Authors	Published Year	Reason for exclusion
SARS-CoV-2 Omicron Symptomatic Infections in Previously Infected or Vaccinated South African Healthcare Workers	Nunes, M. C.; Mbotwe-Sibanda, S.; Baillie, V. L.; Kwatra, G.; Aguas, R.; Madhi, S. A.	2022	unclear/ wrong reinfection definition
Is disease-modifying therapy use in the multiple sclerosis a risk factor during the COVID-19 pandemic? A large cohort study	Ozakbas, S.; Baba, C.; Yavas, I.; Samadzade, U.; Ozdogar, A. T.	2022	no full text
Prevalence of symptoms, comorbidities, and reinfections in individuals infected with Wild-Type SARS-CoV-2, Delta, or Omicron variants: a comparative study in western Mexico	PeÃ±a RodrÃguez, Marcela; HernÃ¡ndez Bello, Jorge; Vega MagaÃ±a, Natali; Viera Segura, Oliver; GarcÃa ChagollÃ¡n, Mariel; Ceja GÃ¡lvez, Hazael Ramiro; Mora Mora, JesÃºs Carlos; RenterÃa Flores, Francisco Israel; GarcÃa GonzÃ¡lez, Octavio Patricio; MuÃ±oz Valle, JosÃ© Francisco	2023	unclear/ wrong reinfection definition
Evidence of SARS-CoV-2 reinfection: analysis of 35,000 subjects and overview of systematic reviews	Pecoraro, V.; Pirotti, T.; Trenti, T.	2023	not Omicron period
SARS-CoV-2 reinfection cases in a household-based prospective cohort in Rio de Janeiro	Penetra, S. L. S.; Santos, H. F. P.; Cristina Resende, P.; Soares Bastos, L.; da Silva, M. F. B.; Pina-Costa, A.; Serrano Lopes, R.; Saboia-Vahia, L.; Caroline Alves de Oliveira, A.; Cavalcante Pereira, E.; Medeiros Filho, F.; Wakimoto, M. D.; Calvet, G. A.; Fuller, T. L.; Whitworth, J.; Smith, C.; Nielsen-Saines, K.; Sa Carvalho, M.; Espindola, O. M.; Guaraldo, L.; Siqueira, M. M.; Brasil, P.	2023	unclear/ wrong reinfection definition
Maternal and neonatal outcomes of Covid-19 re-infection in pregnancy: a national observational study in israel	Peretz, A. C.; Lipschuetz, M.; Guedalia, J.; Calderon-Margalit, R.; Cohen, S.; Walfisch, A.; Yagel, S.; Beharier, O.	2023	no full text

Title	Authors	Published Year	Reason for exclusion
Incidence of SARS-CoV-2 Infection during the Omicron Variant Emergence in Southern Vietnam: Prior Infection versus Third-Dose Vaccination	Phan, T. T.; Nguyen, T. B.; Phung, Q. G. T.; Tran, V. T.; Ho, T. T.; Pho, S. P.; Quach, T. H.; Truong, A. T.; Nguyen, H. T.; Nguyen, T. T.; Nguyen, S. T.	2022	wrong outcomes
Long COVID in Children and Young after Infection or Reinfection with the Omicron Variant: A Prospective Observational Study	Pinto Pereira, Snehal M.; Mensah, Anna; Nugawela, Manjula D.; Stephenson, Terence; Ladhani, Shamez N.; Dalrymple, Emma; Dudley, Jake; McOwat, Kelsey; Simmons, Ruth; Heyman, Isobel; Segal, Terry; Semple, Malcolm G.; Xu, Laila; Shafran, Roz	2023	wrong outcomes
Increased risk of SARS-CoV-2 reinfection associated with emergence of Omicron in South Africa	Pulliam, J. R. C.; van Schalkwyk, C.; Govender, N.; von Gottberg, A.; Cohen, C.; Groome, M. J.; Dushoff, J.; Mlisana, K.; Moultrie, H.	2022	wrong outcomes
Population immunity of natural infection, primary-series vaccination, and booster vaccination in Qatar during the COVID-19 pandemic: an observational study	Qassim, S. H.; Chemaitelly, H.; Ayoub, H. H.; Coyle, P.; Tang, P.; Yassine, H. M.; Al Thani, A. A.; Al-Khatib, H. A.; Hasan, M. R.; Al-Kanaani, Z.; Al-Kuwari, E.; Jeremijenko, A.; Kaleeckal, A. H.; Latif, A. N.; Shaik, R. M.; Abdul-Rahim, H. F.; Nasrallah, G. K.; Al-Kuwari, M. G.; Butt, A. A.; Al-Romaihi, H. E.; Al-Thani, M. H.; Al-Khal, A.; Bertollini, R.; Abu-Raddad, L. J.	2023	not Omicron period
Effects of BA.1/BA.2 subvariant, vaccination and prior infection on infectiousness of SARS-CoV-2 omicron infections	Qassim, Suelen H.; Chemaitelly, Hiam; Ayoub, Houssein H.; AlMukdad, Sawsan; Tang, Patrick; Hasan, Mohammad R.; Yassine, Hadi M.; Al-Khatib, Hebah A.; Smatti, Maria K.; Abdul-Rahim, Hanan F.; Nasrallah, Gheyath K.; Al-Kuwari, Mohamed Ghaith; Al-Khal, Abdullatif; Coyle, Peter; Kaleeckal, Anvar Hassan; Shaik, Riyazuddin Mohammad; Latif, Ali Nizar; Al-Kuwari, Einas; Jeremijenko, Andrew; Butt, Adeel A.	2022	wrong outcomes
The REinfection in COVID-19 Estimation of Risk (RECOVER) study: Reinfection and serology dynamics in a cohort of Canadian healthcare workers	Racine, E.; Boivin, G.; Longtin, Y.; McCormack, D.; Decaluwe, H.; Savard, P.; Cheng, M. P.; Hamelin, M. E.; Carbonneau, J.; Tadount, F.; Adams, K.; Bourdin, B.; Nantel, S.; Gilca, V.; Corbeil, J.; De Serres, G.; Quach-Thanh, C.	2022	not Omicron period

Title	Authors	Published Year	Reason for exclusion
Clinical characteristics of pediatric patients with confirmed SARS-CoV-2 infection who followed rigorous measures during two years of the COVID-19 pandemic in a hospital in Mexico	Ramirez-Cazares, A. C.; Hernandez-Ruiz, Y. G.; Martinez-Longoria, C. A.; Tamez-Gomez, C. E.; Medina-Macias, O.; Trevino-Montalvo, R. G.	2023	unclear/ wrong reinfection definition
Clinical Evolution and Risk Factors in Patients Infected during the First Wave of COVID-19: A Two-Year Longitudinal Study	Rescalvo-Casas, C.; Perez-Tanoira, R.; Villegas, R. F.; Hernando-Gozalo, M.; Seijas-Pereda, L.; Perez-Garcia, F.; Morinigo, H. M.; Gomez-Herruz, P.; Arroyo, T.; Gonzalez, R.; Exposito, C. V.; Lledo Garcia, L.; Cabrera, J. R.; Cuadros-Gonzalez, J.	2023	unclear/ wrong reinfection definition
Characterisation of Omicron Variant during COVID-19 Pandemic and the Impact of Vaccination, Transmission Rate, Mortality, and Reinfection in South Africa, Germany, and Brazil	Ribeiro Xavier, Carolina; Sachetto Oliveira, Rafael; da Fonseca Vieira, VinÃcius; Lobosco, Marcelo; Weber Dos Santos, Rodrigo	2022	wrong study design
Incidence of COVID-19 reinfection among Midwestern healthcare employees	Rivelli, Anne; Fitzpatrick, Veronica; Blair, Christopher; Copeland, Kenneth; Richards, Jon	2022	not Omicron period
Impact of Severe Acute Respiratory Syndrome Coronavirus 2 Variants on Inpatient Clinical Outcome	Robinson, Matthew L.; Morris, C. Paul; Betz, Joshua F.; Zhang, Yifan; Bollinger, Robert; Wang, Natalie; Thiemann, David R.; Fall, Amary; Eldesouki, Raghda E.; Norton, Julie M.; Gaston, David C.; Forman, Michael; Luo, Chun Huai; Zeger, Scott L.; Gupta, Amita; Garibaldi, Brian T.; Mostafa, Heba H.	2023	wrong outcomes
Early SARS-CoV-2 Reinfections Involving the Same or Different Genomic Lineages, Spain	Rodriguez-Grand, C.; Estevez, A.; Palomino-Cabrer, R.; Molero-Salina, A.; Penas-Utrill, D.; Herranz, M.; Sanz-Pere, A.; Alcala, L.; Veintimilla, C.; Catalan, P.; Martinez-Laperch, C.; Alonso, R.; Munoz, P.; Perez-Lag, L.; De Viedma, D. G.	2023	unclear/ wrong reinfection definition
Rapid Increase in Suspected SARS-CoV-2 Reinfections, Clark County, Nevada, USA, December 2021	Ruff, Jeanne; Zhang, Ying; Kappel, Matthew; Rathi, Sfurti; Watkins, Kellie; Zhang, Lei; Lockett, Cassius	2022	wrong outcomes

Title	Authors	Published Year	Reason for exclusion
Cases of SARS-CoV-2 reinfection with Omicron BA.2 post breakthrough infection with Delta and Kappa variants	Sahay, R. R.; Patil, D. Y.; Sapkal, G. N.; Shete, A. M.; Yadav, P. D.	2023	wrong study design
COVID-19 reinfection or relapse? A retrospective multicenter cohort study from Iran	Salehi, M.; Seyedalinaghi, S.; Darazam, I. A.; Tabarsi, P.; Rabiei, M. M.; Hatami, F.; Ghadimi, S.; Koochak, H. E.; Veisi, P.; Ghiasvand, F.; Asadollahi-Amin, A.	2021	not Omicron period
SARS-CoV-2 re-infection rate in Iranian COVID-19 cases within one-year follow-up	Salehi-Vaziri, Mostafa; Pouriayevali, Mohammad Hassan; Fotouhi, Fatemeh; Jalali, Tahmineh; Banifazl, Mohammad; Farahmand, Behrokh; Sadat Larijani, Mona; Ahmadi, Zahra; Fereydouni, Zahra; Tavakoli, Mahsa; Karami, Afsaneh; Azad-Manjiri, Sanam; Yektay Sanati, Parastoo; Dahmardeh, Sarah; Nemati, Amir Hesam; Sajadi, Marzyie; Kashanian, Setareh; Ramezani, Amitis	2021	not Omicron period
Senjuti Saha: advocate for equity in global health research	Samarasekera, Udani	2022	wrong study design
[Reinfection by the Omicron variant in patients previously infected with the Delta variant of the SARS-CoV-2 coronavirus: An increasingly frequent reality in Primary Care]	Sanchez-Varela, N.; Cinza-Sanjurjo, S.; Portela-Romero, M.	2022	wrong study design
Necessity of Coronavirus Disease 2019 (COVID-19) Vaccination in Persons Who Have Already Had COVID-19	Shrestha, Nabin K.; Burke, Patrick C.; Nowacki, Amy S.; Terpeluk, Paul; Gordon, Steven M.	2022	wrong outcomes
Clinical course of SARS-CoV-2 infections of paediatric patients with cystic fibrosis- a single retrospective centre experience	Sieber, J.; Strasser, N.; Schmidthaler, K.; Dehlink, E.; Gaupmann, R.; Szepfalusi, Z.; Gruber, S.	2023	no full text

Title	Authors	Published Year	Reason for exclusion
Contamination by Covid-19 in professionals of a reference hospital in Para's state	Silva, Elaine AbrahÃ£o Dias; Chaves, FÃ¡bio JosÃ© Maciel; Cals, Rita de CÃ¡ssia Frota Vieira; Silva, Andreia do Carmo Gomes da; GonÃ§alves, Rodrigo Dantas; Magno, Lina Cristina de Paula	2023	wrong language
Prevalence and Predictors of COVID-19 Long-Term Symptoms: A Cohort Study from the Amazon Basin	Silva, K. M.; Freitas, D. C. A.; Medeiros, S. S.; Miranda, L. V. A.; Carmo, J. B. M.; Silva, R. G.; Becker, L. L.; Abreu, E. S.; Buranello, L.; Souza, M. S. M.; Nadruz, W.; Fernandes-Silva, M. M.; Maguire, J. H.; Toledo-Cornell, C.; Silvestre, O. M.	2023	not Omicron period
Clinical and Anamnestic Characteristics, Cardiovascular Pharmacotherapy and Long-term Outcomes in Multimorbid Patients after COVID-19	Smirnov, A. A.; Loukianov, M. M.; Martsevich, S. Yu Pulin A. A.; Kutishenko, N. P.; Andreenko, E. Yu Voronina V. P.; Dindikova, V. A.; Dmitrieva, N. A.; Kudryavtseva, M. M.; Lerman, O. V.; Makoveeva, A. N.; Okshina, E. Yu Maltseva A. A.; Belova, E. N.; Klyashtorniy, V. G.; Kudryashov, E. V.; Karpov, O. E.; Drapkina, O. M.	2022	wrong language
Clinical Characteristics of Patients Who Contracted the SARS-CoV-2 Omicron Variant from an Outbreak in a Single Hospital	Sohn, Yu Jin; Shin, Pyo Jin; Oh, Won Sup; Kim, Eunmi; Kim, Yeojin; Kim, Young Keun	2022	unclear/ wrong reinfection definition
Surveillance of Severe Acute Respiratory Syndrome Coronavirus 2 and Variants Using Digital Droplet Polymerase Chain Reaction at a Large University and Healthcare System in California	Stafylis, C.; Pernet, O.; Hernandez-Tamayo, C.; Kovacs, A.; Emerson, J.; Ward, P. M.; Van Orman, S.; Gilliland, F.; Conti, D.; Weisenhaus, M.; Ghanem-Uzqueda, A.; Yepez, D.; Stellar, S.; Tadanki, A. P.; Max, J.; Fottrell, H.; Ong, E.; Navarro, S.; Moses, K.; Akaolisa, M.; Hosseini, B.; Sunesara, S.; Wang, Y.; Strum, E.; Casagrande, Y.; Arenas, N.; Williams, C.; Thomas, P.; Chu, T.; Hu, H.; Klausner, J. D.	2023	wrong outcomes
Outcomes associated with SARS-CoV-2 reinfection in individuals with natural and hybrid immunity	Suleyman, G.; Fadel, R.; Patel, K.; Shadid, A. M.; Stuart, H. B. C.; Kattula, M.; Janis, A.; Maki, M.; Chao, S.; Alangaden, G.; Brar, I.	2023	wrong outcomes
Outcomes associated with SARS-CoV-2 reinfection in individuals with natural and hybrid immunity	Suleyman, Geehan; Fadel, Raef; Patel, Kunj; Shadid, Al Muthanna; Stuart, Haim Bernardo Cotlear; Kattula, Michael; Janis, Andrea; Maki, Mohamed; Chao, Shing; Alangaden, George; Brar, Indira	2023	wrong outcomes

Title	Authors	Published Year	Reason for exclusion
Antibody titer levels and the effect on subsequent SARS-CoV-2 infection in a large US-based cohort. (Special Issue: Quantitative biology, biotechnology and bioengineering.)	Sullivan, A.; Alfego, D.; Hu, PingSha; Gillim, L.; Grover, A.; Garcia, C.; Cohen, O.; Letovsky, S.	2023	not Omicron period
Antibody titer levels and the effect on subsequent SARS-CoV-2 infection in a large US-based cohort	Sullivan, Adam; Alfego, David; Hu, Pingsha; Gillim, Laura; Grover, Ajay; Garcia, Chris; Cohen, Oren; Letovsky, Stan	2023	not Omicron period
Rapidly shifting immunologic landscape and severity of SARS-CoV-2 in the Omicron era in South Africa	Sun, K.; Tempia, S.; Kleynhans, J.; von Gottberg, A.; McMorrow, M. L.; Wolter, N.; Bhiman, J. N.; Moyes, J.; Carrim, M.; Martinson, N. A.; Kahn, K.; Lebina, L.; du Toit, J. D.; Mkhencele, T.; Viboud, C.; Cohen, C.; Buys, A.; de Gouveia, L.; du Plessis, M.; Gomez-Olive, F. X.; Kgasago, K. P.; Kotane, R.; Moloantoa, T.; Tollman, S.; Wafawanaka, F.	2023	unclear/ wrong reinfection definition
Infectiousness of SARS-CoV-2 breakthrough infections and reinfections during the Omicron wave	Tan, S. T.; Kwan, A. T.; Rodriguez-Barraquer, I.; Singer, B. J.; Park, H. J.; Lewnard, J. A.; Sears, D.; Lo, N. C.	2023	unclear/ wrong reinfection definition
Demographic and Clinical Characteristics of Suspect SARS-CoV-2 Reinfection Cases in Los Angeles County from March 10 to June 1, 2021: A Crosssectional Study of Case Interview Data	Thompson, J.; Masai, L.; Khalid, N.; Chun, K.; Khuu, D.; Alkhoudairy, N. M.; Griffin, J. B.	2021	not Omicron period
Clinical course of SARS-CoV-2 infection and recovery in lung transplant recipients	Trindade, A. J.; Chapin, K. C.; Gannon, W. D.; Hoy, H.; Demarest, C. T.; Lambright, E. S.; McPherson, K. A.; Norfolk, S. G.; Robbins, I. M.; Bacchetta, M.; Erasmus, D. B.; Shaver, C. M.	2022	unclear/ wrong reinfection definition
Predictors of Recurrent Laboratory-Confirmed Symptomatic SARS-CoV-2 Infections in a Cohort of Healthcare Workers	Trujillo, X.; Mendoza-Cano, O.; Rios-Silva, M.; Huerta, M.; Guzman-Esquivel, J.; Benites-Godinez, V.; Lugo-Radillo, A.; Bricio-Barrios, J. A.; Cardenas-Rojas, M. I.; Rios-Bracamontes, E. F.; Ortega-Macias, V. M.; Ruiz-Montes de Oca, V.; Murillo-Zamora, E.	2023	not Omicron period

Title	Authors	Published Year	Reason for exclusion
Genomic evidence of SARS-CoV-2 reinfection cases in southern Brazil	Varela, A. P. M.; Sant'Anna, F. H.; Dos Santos, A. V.; Prichula, J.; Comerlato, J.; Dos Santos, G. T.; Wendland, E.	2023	not Omicron period
Omicron-Associated Changes in Severe Acute Respiratory Syndrome Coronavirus 2 (SARS-CoV-2) Symptoms in the United Kingdom	Vihta, K. D.; Pouwels, K. B.; Peto, T. E. A.; Pritchard, E.; House, T.; Studley, R.; Rourke, E.; Diamond, I.; Clifton, D. A.; Matthews, P. C.; Stoesser, N.; Eyre, D. W.; Walker, A. S.; Thomas, T.; Cook, D.; Ayoubkhani, D.; Black, R.; Felton, A.; Crees, M.; Jones, J.; Lloyd, L.; Sutherland, E.; Crook, D.; Wei, J.; Howarth, A.; Doherty, G.; Kavanagh, J.; Chau, K. K.; Stephanie, H. B.; Ebner, D.; Martins Ferreira, L.; Christott, T.; Marsden, B. D.; Dejnirattisai, W.; Mongkolsapaya, J.; Cameron, S.; Tamblin-Hopper, P.; Wolna, M.; Brown, R.; Hoosdally, S.; Cornall, R.; Jones, Y.; Stuart, D. I.; Screaton, G.; Lythgoe, K.; Bonsall, D.; Golubchik, T.; Fryer, H.; Bell, J.; Paddon, K.; James, T.; Newton, J.; Robotham, J.; Birrell, P.; Jordan, H.; Sheppard, T.; Athey, G.; Moody, D.; Curry, L.; Brereton, P.; Jarvis, I.; Godsmark, A.; Morris, G.; Mallick, B.; Eeles, P.; Hay, J.; Lee, J.; Sean, W.; Evans, T.; Bloemberg, L.; Allison, K.; Pandya, A.; Davis, S.; Conway, D. I.; Macleod, M.; Cunningham, C.	2023	unclear/ wrong reinfection definition
Hybrid Immunity Provides Protective Advantage Over Vaccination or Prior Remote Coronavirus Disease 2019 Alone	Virk, A.; Johnson, M. G.; Roellinger, D. L.; Scott, C. G.; Sampathkumar, P.; Breeher, L. E.; Swift, M.	2023	wrong outcomes
The evolving landscape of COVID-19 and post-COVID condition in patients with chronic lymphocytic leukemia: A study by ERIC, the European research initiative on CLL	Visentin, A.; Chatzikonstantinou, T.; Scarfo, L.; Kapetanakis, A.; Demosthenous, C.; Karakatsoulis, G.	2023	unclear/ wrong reinfection definition
Assessment of SARS-CoV-2 Reinfection 1 Year After Primary Infection in a Population in Lombardy, Italy	Vitale, JosÃ¨; Mumoli, Nicola; Clerici, Pierangelo; De Paschale, Massimo; Evangelista, Isabella; Cei, Marco; Mazzone, Antonino	2021	not Omicron period

Title	Authors	Published Year	Reason for exclusion
Impact of prior SARS-CoV-2 infection and COVID-19 vaccination on the subsequent incidence of COVID-19: A multicentre prospective cohort study among UK healthcare workers-the SIREN (Sarscov2 Immunity & REinfection EvaluatioN) study protocol	Wallace, S.; Hall, V.; Charlett, A.; Kirwan, P. D.; Cole, M.; Gillson, N.; Atti, A.; Timeyin, J.; Foulkes, S.; Taylor-Kerr, A.; Andrews, N.; Shrotri, M.; Rokadiya, S.; Oguti, B.; Vusirikala, A.; Islam, J.; Zambon, M.; Brooks, T. J. G.; Ramsay, M.; Brown, C. S.; Chand, M.; Hopkins, S.	2022	wrong study design
SARS-CoV-2 Reinfection With Different SARS-CoV-2 Variants in Children, Ohio, United States	Wang, H.; Wright, T.; Everhart, K.; Oyeniran, S. J.; Mejias, A.; Leber, A. L.	2023	wrong outcomes
Impact of Vaccination, Prior Infection, and Therapy on Omicron Infection and Mortality	Wang, X.; Zein, J.; Ji, X.; Lin, D. Y.	2023	wrong outcomes
Impact of vaccination, prior infection, and therapy on omicron infection and mortality	Wang, XiaoFeng; Zein, Joe; Ji, XinGe; Lin, DanYu	2022	wrong outcomes
Effect of the incremental protection of previous infection against Omicron infection among individuals with a hybrid of infection- and vaccine-induced immunity: a population-based cohort study in Canada	Wu, S.; Li, Y.; Mishra, S.; Bodner, K.; Baral, S.; Kwong, J. C.; Wei, X.	2023	wrong outcomes
SARS-CoV-2 in Malaysia: A surge of reinfection during the predominantly Omicron period	Yang, S. L.; Teh, H. S.; Suah, J. L.; Husin, M.; Hwong, W. Y.	2022	not Omicron period

Title	Authors	Published Year	Reason for exclusion
The omicron variant reinfection risk among individuals with a previous SARS-CoV-2 infection within one year in Shanghai, China: a cross-sectional study	Ye, ChuChu; Zhang, Ge; Zhang, AnRan; Xin, HuaLei; Wu, Kang; Li, ZhongJie; Jia, YiLin; Hao, LiPeng; Xue, CaoYi; Wang, YuanPing; Xu, HongMei; Zhu, WeiPing; Zhou, YiXin	2023	unclear/ wrong reinfection definition
Effect of genetics polymorphisms on reinfection with COVID-19 and progression severity	Yessenbayeva, A. A.; Massabayeva, M. R.; Apsalikov, B. A.; Zholambayeva, Z. S.; Khamitova, M. O.; Khamidullina, Z. G.; Kassym, L. T.	2023	not Omicron period
A Case-Control Study Investigating Household, Community, and Clinical Risk Factors Associated with Multisystem Inflammatory Syndrome in Children (MIS-C) after SARS-CoV-2 Infection	Zambrano, L. D.; Wu, M. J.; Martin, L. M.; Malloch, L.; Newhams, M. M.; Beth Son, M.; Sanders, C.; Patterson, K.; Halasa, N. B.; Fitzgerald, J. C.; Leroue, M.; Hall, M.; Irby, K.; Rowan, C. M.; Wellnitz, K.; Loftis, L. L.; Bradford, T. T.; Staat, M. A.; Babbit, C.; Carroll, C. L.; Pannaraj, P. S.; Kong, M.; Chou, J.; Patel, M. M.; Randolph, A. G.; Campbell, A. P.; Hobbs, C. V.	2022	no full text
COVID-19 re-infection in Shahroud, Iran: a follow-up study	Zare, Fariba; Teimouri, Maryam; Khosravi, Ahmad; Rohani-Rasaf, Marzieh; Chaman, Reza; Hosseinzadeh, Ali; Jamali Atergeleh, Hozhabr; Binesh, Ehsan; Emamian, Mohammad Hassan	2021	not Omicron period
The Infection of Healthcare Workers and the Reinfection of Patients by Omicron Variant - Jiangsu Province, China, December 2022 to January 2023	Zhang, Chuanmeng; Guo, Ting; Zhang, Lei; Gu, Aiqin; Ye, Jun; Lin, Mei; Chu, Ming; Zhu, Fengcai; Zhu, Li	2023	unclear/ wrong reinfection definition
Management strategies for pediatric wards of designated hospitals for omicron variant BA.5.1.3 infection in south area of Hainan	Zhang, Hua; Chen, HaiDan; Chen, ZeYan; Qiu, FeiQin; Gao, Lin; Zhu, Hong; Wang, XiaoLi; Wu, XiaYing	2023	no full text

Title	Authors	Published Year	Reason for exclusion
The Correlation Between Triglyceride-Glucose Index and SARS-CoV-2 RNA Re-Positive in Discharged COVID-19 Patients	Zheng, Yufen; Wang, Jing; Ding, Xianhong; Chen, Shiyong; Li, Jun; Shen, Bo	2022	not Omicron period

Table S3: Cumulative incidence of SARS-CoV-2 reinfections by comorbidity status

Study	comorbidity	Number of reinfections	Number of primary infections	Cumulative incidence (%)
Abuhasira 2023	diabetes	13154	174558	07.53 (07.42 – 07.66)
Abuhasira 2023	COPD	4744	62134	07.64 (07.44 – 07.84)
Abuhasira 2023	Asthma	12989	140772	09.23 (09.08 – 09.37)
Abuhasira 2023	Chronic kidney failure	3927	55315	07.10 (06.90 – 07.31)
Abuhasira 2023	Hypertension	20645	287347	07.18 (07.09 – 07.28)
Abuhasira 2023	Ischemic heart disease	5523	86523	06.38 (06.23 – 06.54)
Abuhasira 2023	Chronic heart failure	2623	36657	07.16 (06.90 – 07.41)
Abuhasira 2023	Obesity	57164	660638	08.65 (08.59 – 08.72)
Abuhasira 2023	Lung cancer	173	2986	05.79 (05.03 – 06.66)
Abuhasira 2023	Malignancies	5123	76476	06.70 (06.53 – 06.87)
Abuhasira 2023	Cerebrovascular event	3985	55626	07.16 (06.96 – 07.37)
Abuhasira 2023	Immunocompromised	3981	43575	09.14 (08.88 – 09.40)
Abuhasira 2023	entire study population	111417	1280649	08.70 (08.65 – 08.75)
Jang 2023	Immunocompromised	2620	447934	00.58 (00.56 – 00.61)
Jang 2023	entire study population	154924	17389037	00.89 (00.89 – 00.90)
Malhotra 2022	any comorbidity	197	710	27.75 (24.93 – 30.75)
Malhotra 2022	entire study population	1007	3545	28.41 (27.11 – 29.73)

Table S4: Data on asymptomatic infections

Study	Asymptomatic infections (%)	Country	Study population
Malhotra 2022	4.37	India	Healthcare workers
Cohen 2023	11.77	Israel	Convalescent plasma donors
Yu 2023	32.43	China	General population
Cai 2023	92.50	China	General population

Table S5: Percentage of reinfections progressing to severe disease

Study	Number of reinfection cases progressing to severe disease	Total number of reinfections	Severe disease (%)	Country	Study population
Sacco 2022	1390	163468	0.85	Italy	General population
Vicentini 2023	34	3243	1.05	Italy	General population
Chemaitelly 2022	4	7995	0.05	Qatar	General population
Yu 2023	9	148	6.08	China	General population

Table S6: Case fatality rate reported in included studies

Study	Number of deaths Reinfections	Number of reinfections	Case fatality rate (per cent)	Criteria
Abuhasira 2023	151	111,417	0.14	Death reported to the Israeli Ministry of Health as COVID-19 related death (no reference)
Chemaitelly 2022	27	7,995	0.34	All deaths were non-COVID-related
Lee 2023	133	71,106	0.19	Death after 28 days of follow-up, after excluding foreign personnel, as reported by the COVID-19 Patient Management Information System (Central Disease Control Headquarters)
Ma 2023	6411	2,701,234	0.24	Six jurisdictions provided only deaths with COVID-19 as a listed underlying or probable cause. The remaining 11 jurisdictions also provided deaths from natural causes or without other evident causes that occurred ≤30 days of positive specimen collection (eight) or some other specified time window (42 days, 45 days, and 60 days).
Vicentini 2023	1	3,243	0.00	Death occurring within 90 days from diagnosis and fulfilled the criteria for reporting COVID-19 as the main cause of death

Table S7a: Age as a risk factor for SARS-CoV-2 reinfections: adjusted and unadjusted hazard ratios, odds ratio and risk ratios reported in included studies

Study	Age group (years)	Measure of effect	Estimate	95%LCI	95%UCI	Reference age group (years)
Abuhasira 2023	65-79	adjusted hazard ratio	0.79	0.77	0.81	16-64
Abuhasira 2023	80+	adjusted hazard ratio	0.95	0.92	0.99	16-64
Cegolon 2023	≥60	adjusted hazard ratio	0.53	0.38	0.74	<30
Cegolon 2023	30-39	adjusted hazard ratio	1.11	0.88	1.41	<30
Cegolon 2023	40-49	adjusted hazard ratio	1.13	0.90	1.43	<30
Cegolon 2023	50-59	adjusted hazard ratio	0.85	0.67	1.06	<30
Jang 2023	12-17	adjusted hazard ratio	2.00	1.95	2.05	40-59
Jang 2023	5-11	adjusted hazard ratio	2.20	2.15	2.26	40-59
Jang 2023	0-4	adjusted hazard ratio	1.13	1.10	1.16	40-59
Jang 2023	18-39	adjusted hazard ratio	1.70	1.67	1.72	40-59
Jang 2023	60-74	adjusted hazard ratio	1.03	1.01	1.06	40-59
Jang 2023	75+	adjusted hazard ratio	1.16	1.12	1.20	40-59
Eythorsson 2022	≤17	adjusted odds ratio	0.81	0.66	0.98	18-29
Eythorsson 2022	≥75	adjusted odds ratio	0.22	0.08	0.61	18-29
Eythorsson 2022	30-49	adjusted odds ratio	0.79	0.66	0.95	18-29
Eythorsson 2022	50-74	adjusted odds ratio	0.32	0.24	0.44	18-29
Lee 2023	0-17	adjusted odds ratio	0.77	0.75	0.78	18-59
Lee 2023	60-74	adjusted odds ratio	0.98	0.95	1.01	18-59
Lee 2023	75+	adjusted odds ratio	0.86	0.79	0.86	18-59
Ye 2023	10-19	adjusted odds ratio	0.66	0.23	1.95	≥80
Ye 2023	0-9	adjusted odds ratio	0.16	0.04	0.76	≥80
Ye 2023	20-29	adjusted odds ratio	1.09	0.51	2.32	≥80
Ye 2023	30-39	adjusted odds ratio	2.03	1.00	4.15	≥80
Ye 2023	40-49	adjusted odds ratio	1.16	0.55	2.43	≥80
Ye 2023	50-59	adjusted odds ratio	0.90	0.43	1.90	≥80
Ye 2023	60-69	adjusted odds ratio	0.82	0.37	1.82	≥80
Ye 2023	70-79	adjusted odds ratio	0.64	0.26	1.62	≥80

Study	Age group (years)	Measure of effect	Estimate	95%LCI	95%UCI	Reference age group (years)
Yu 2023	>80	adjusted odds ratio	0.86	0.16	3.64	41-60
Yu 2023	≤20	adjusted odds ratio	0.64	0.14	2.10	41-60
Yu 2023	21-40	adjusted odds ratio	1.34	0.74	2.47	41-60
Yu 2023	61-80	adjusted odds ratio	0.57	0.22	1.36	41-60
Cohen 2023	≥35	adjusted risk ratio	0.73	0.45	1.16	18-34
Abuhasira 2023	65-79	unadjusted hazard ratio	0.77	0.75	0.78	16-64
Abuhasira 2023	80+	unadjusted hazard ratio	1.09	1.05	1.13	16-64
Eythorsson 2022	≤17	unadjusted odd ratio	0.68	0.58	0.81	18-29
Cai 2023	≥60	unadjusted odds ratio	0.64			18-59
Cai 2023	0-17	unadjusted odds ratio	0.55			18-59
Eythorsson 2022	≥75	unadjusted odds ratio	0.24	0.15	0.45	18-29
Eythorsson 2022	30-49	unadjusted odds ratio	0.82	0.72	0.94	18-29
Eythorsson 2022	50-74	unadjusted odds ratio	0.35	0.29	0.43	18-29
Lee 2023	0-17	unadjusted odds ratio	1.63	1.61	1.66	18-59
Lee 2023	60-74	unadjusted odds ratio	0.80	0.78	0.82	18-59
Lee 2023	75+	unadjusted odds ratio	0.93	0.89	0.96	18-59
Ye 2023	10-19	unadjusted odds ratio	0.67			20-29
Ye 2023	≥80	unadjusted odds ratio	1.10			20-29
Ye 2023	0-9	unadjusted odds ratio	0.21			20-29
Ye 2023	30-39	unadjusted odds ratio	1.58			20-29
Ye 2023	40-49	unadjusted odds ratio	0.97			20-29
Ye 2023	50-59	unadjusted odds ratio	0.78			20-29
Ye 2023	60-69	unadjusted odds ratio	0.75			20-29
Ye 2023	70-79	unadjusted odds ratio	0.63			20-29
Cohen 2023	≥35	unadjusted risk ratio	0.61	0.39	0.97	18-34

Table S7b: Protection by COVID vaccines from SARS-CoV-2 reinfections: adjusted and unadjusted hazard ratios, odds ratios, and risk ratios

Study	Number of vaccine doses	Measure of effect	Estimate	95%LCI	95%UCI	Number of vaccine doses in reference group
Cegolon 2023	4	adjusted hazard ratio	0.08	0.05	0.14	0
Jang 2023	3	adjusted hazard ratio	0.20	0.07	0.53	0
Cegolon 2023	3	adjusted hazard ratio	0.24	0.20	0.28	0
Cegolon 2023	2	adjusted hazard ratio	0.49	0.38	0.63	0
Abuhasira 2023	2	adjusted hazard ratio	0.54	0.53	0.55	0
Jang 2023	2	adjusted hazard ratio	0.54	0.49	0.59	0
Abuhasira 2023	≥3	adjusted hazard ratio	0.80	0.78	0.81	0
Cegolon 2023	1	adjusted hazard ratio	0.84	0.56	1.25	0
Abuhasira 2023	1	adjusted hazard ratio	0.92	0.9	0.93	0
Jang 2023	1	adjusted hazard ratio	0.94	0.92	0.96	0
Lee 2023	3	adjusted odds ratio	0.26	0.25	0.26	0
Lee 2023	4	adjusted odds ratio	0.47	0.43	0.51	0
Lee 2023	2	adjusted odds ratio	0.52	0.50	0.53	0
Ye 2023	≥2	adjusted odds ratio	0.58	0.41	0.81	0 or 1
Yu 2023	2	adjusted odds ratio	0.72	0.31	1.68	0 or 1
Ye 2023	2	adjusted odds ratio	0.74	0.528	1.04	0 or 1
Yu 2023	≥2	adjusted odds ratio	0.84	0.39	1.90	0 or 1
Lee 2023	1	adjusted odds ratio	0.99	0.94	1.05	0
Eythorsson 2022	≥2	adjusted odds ratio	1.42	1.13	1.78	0 or 1
Cohen 2023	≥3	adjusted risk ratio	0.28	0.10	0.75	0
Cohen 2023	2	adjusted risk ratio	0.44	0.23	0.82	0
Cohen 2023	1	adjusted risk ratio	0.55	0.31	0.99	0
Abuhasira 2023	≥3	unadjusted hazard ratio	0.25	0.24	0.25	0
Abuhasira 2023	2	unadjusted hazard ratio	0.51	0.5	0.52	0

Study	Number of vaccine doses	Measure of effect	Estimate	95%LCI	95%UCI	Number of vaccine doses in reference group
Malhotra 2022	1	unadjusted hazard ratio	0.89	0.65	1.21	0
Malhotra 2022	1	unadjusted hazard ratio	1.05	0.77	1.42	0
Malhotra 2022	2	unadjusted hazard ratio	1.19	0.93	1.52	0
Malhotra 2022	2	unadjusted hazard ratio	1.19	0.93	1.52	0
Abuhasira 2023	1	unadjusted hazard ratio	1.26	1.24	1.28	0
Lee 2023	3	unadjusted odds ratio	0.34	0.33	0.35	0
Lee 2023	2	unadjusted odds ratio	0.63	0.62	0.64	0
Ye 2023	≥2	unadjusted odds ratio	0.75	0.55	1.03	0 or 1
Ye 2023	2	unadjusted odds ratio	0.87	0.63	1.20	0 or 1
Eythorsson 2022	≥2	unadjusted odds ratio	0.92	0.81	1.05	0 or 1
Lee 2023	4	unadjusted odds ratio	1.04	0.95	1.13	0
Lee 2023	1	unadjusted odds ratio	1.16	1.09	1.22	0
Cohen 2023	≥3	unadjusted risk ratio	0.30	0.11	0.79	0
Cohen 2023	2	unadjusted risk ratio	0.44	0.24	0.82	0
Cohen 2023	1	unadjusted risk ratio	0.56	0.32	0.99	0

Table S7c: Comorbidities as a risk of SARS-CoV-2 reinfections: Adjusted and unadjusted hazard ratios

Study	Comorbidity	Measure of effect	Estimate	95% LCI	95% UCI
Abuhasira 2023	asthma	adjusted hazard ratio	1.09	1.07	1.11
Abuhasira 2023	chronic heart failure	adjusted hazard ratio	1.04	0.99	1.08
Abuhasira 2023	chronic kidney failure	adjusted hazard ratio	1.16	1.12	1.21
Abuhasira 2023	COPD	adjusted hazard ratio	0.99	0.96	1.02
Abuhasira 2023	diabetes	adjusted hazard ratio	0.92	0.9	0.94
Abuhasira 2023	history of stroke of TIA	adjusted hazard ratio	1.18	1.14	1.22
Abuhasira 2023	hypertension	adjusted hazard ratio	1.08	1.06	1.10
Abuhasira 2023	immunocompromised	adjusted hazard ratio	1.27	1.23	1.32
Abuhasira 2023	ischaemic heart disease	adjusted hazard ratio	1.06	1.03	1.09
Abuhasira 2023	obesity	adjusted hazard ratio	1.01	1.00	1.03
Abuhasira 2023	asthma	unadjusted hazard ratio	1.09	1.07	1.11
Abuhasira 2023	chronic heart failure	unadjusted hazard ratio	1.11	1.07	1.15
Abuhasira 2023	chronic kidney failure	unadjusted hazard ratio	1.09	1.06	1.13
Abuhasira 2023	COPD	unadjusted hazard ratio	1.02	0.99	1.05
Abuhasira 2023	diabetes	unadjusted hazard ratio	0.98	0.96	1.00
Abuhasira 2023	history of stroke of TIA	unadjusted hazard ratio	1.08	1.05	1.11
Abuhasira 2023	hypertension	unadjusted hazard ratio	0.98	0.96	0.99
Abuhasira 2023	immunocompromised	unadjusted hazard ratio	1.24	1.21	1.28
Abuhasira 2023	ischaemic heart disease	unadjusted hazard ratio	0.93	0.91	0.96
Abuhasira 2023	obesity	unadjusted hazard ratio	1.03	1.02	1.04
Jang 2023	immunocompromised	adjusted hazard ratio	1.23	1.18	1.28
Jang 2023	LTCF residents	adjusted hazard ratio	2.34	2.26	2.43
Malhotra 2022	any comorbidity	adjusted hazard ratio	1.04	0.88	1.23

COPD = chronic obstructive pulmonary disease; TIA = transient ischaemic attack; LTCF = long-term care facility

Table S7d: Sex as a risk factor of SARS-CoV-2 reinfections: Adjusted and unadjusted hazard ratios, odds ratios and unadjusted risk ratio in females compared to males

Study	Sex	Measure of effect	Estimate	95%LCI	95%UCI
Jang 2023	female	adjusted hazard ratio	1.18	1.17	1.2
Cegolon 2023	female	adjusted hazard ratio	1.19	1.02	1.37
Abuhasira 2023	female	adjusted hazard ratio	1.43	1.41	1.45
Ye 2023	female	adjusted odds ratio	0.732	0.557	0.961
Lee 2023	female	adjusted odds ratio	0.98	0.96	0.99
Yu 2023	female	adjusted odds ratio	2.19	1.29	3.83
Abuhasira 2023	female	unadjusted hazard ratio	1.32	1.30	1.33
Ye 2023	female	unadjusted odds ratio	0.76	0.57	1.02
Eythorsson 2022	female	unadjusted odds ratio	0.97	0.87	1.09
Lee 2023	female	unadjusted odds ratio	0.97	0.95	0.98
Cai 2023	female	unadjusted odds ratio	1.03	0.61	1.74
Cohen 2023	female	unadjusted risk ratio	1.09	0.65	1.85

Figure S1a: Cumulative incidence of SARS-CoV-2 reinfections during the Omicron period (n = 28)

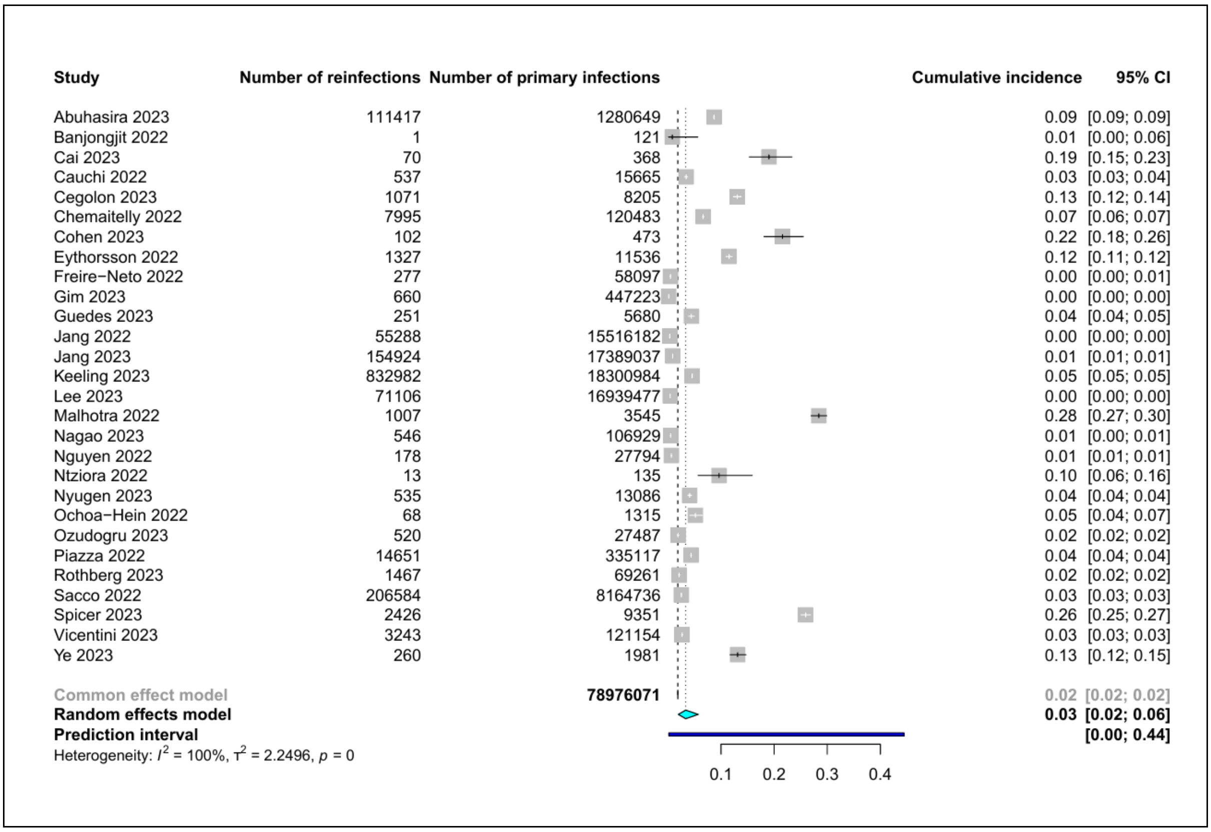

Figure S1b: Cumulative incidence of SARS-CoV-2 reinfections during the Omicron period by definition of minimum interval between the two infections (n = 28)

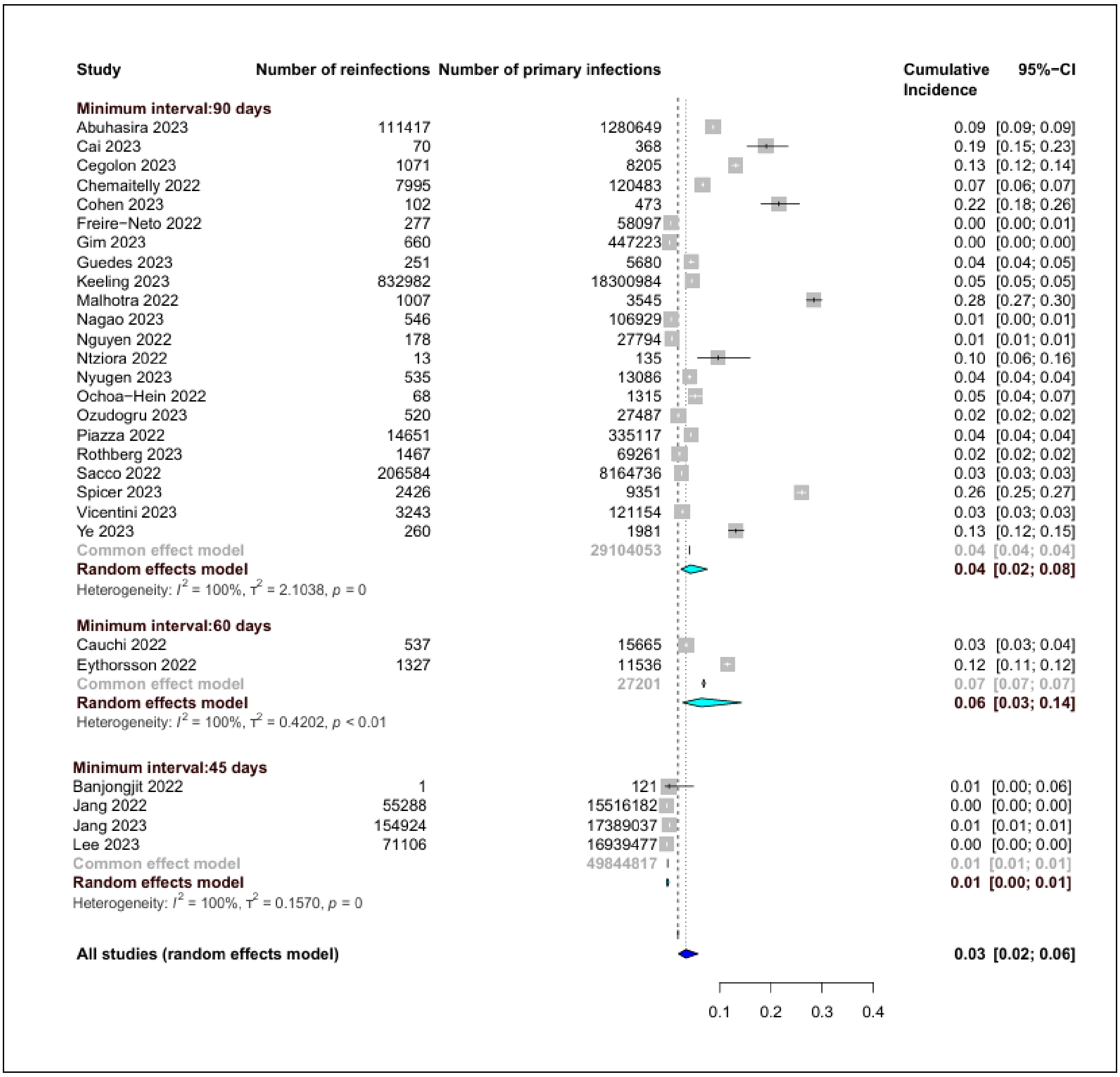

Figure S1c: Cumulative incidence of SARS-CoV-2 reinfections by population type (n = 28)

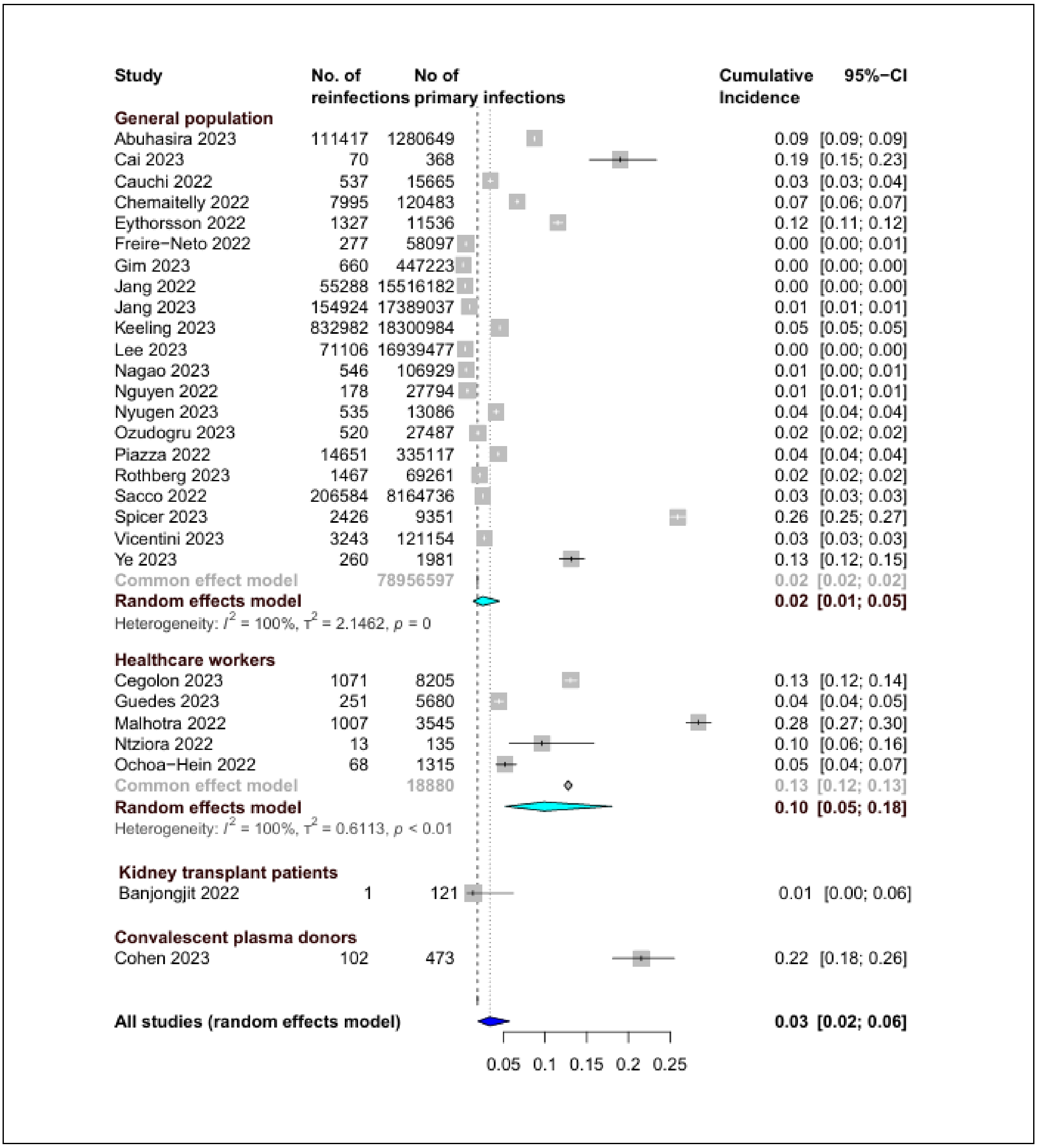

Figure S1d: Cumulative incidence of SARS-CoV-2 reinfections by age (n = 16)

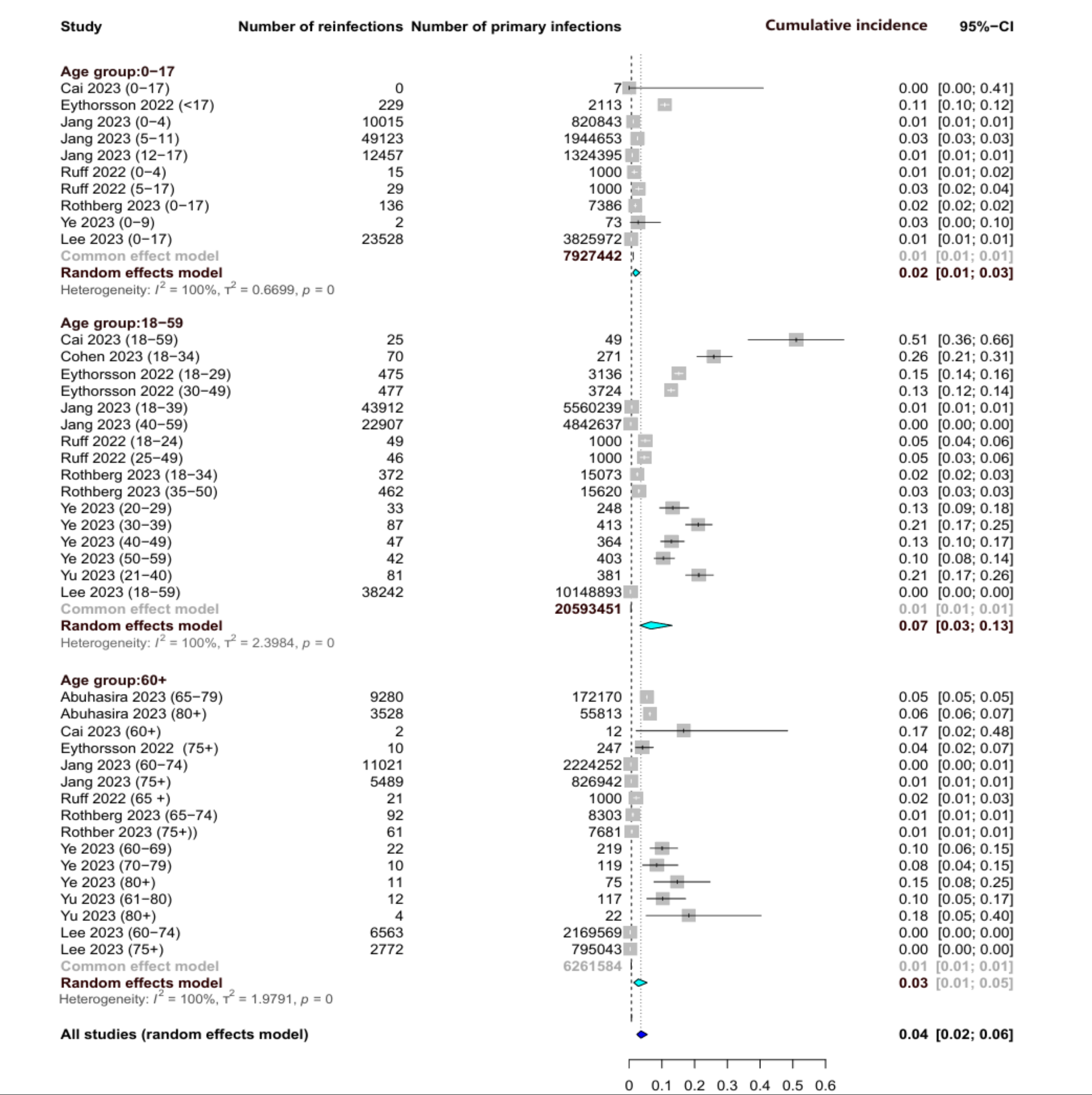

All studies = all age groups from all studies included in the age analysis (n = 16)

Figure S1e: Cumulative incidence (incidence proportion) of SARS-CoV-2 reinfections by vaccination status (n = 8)

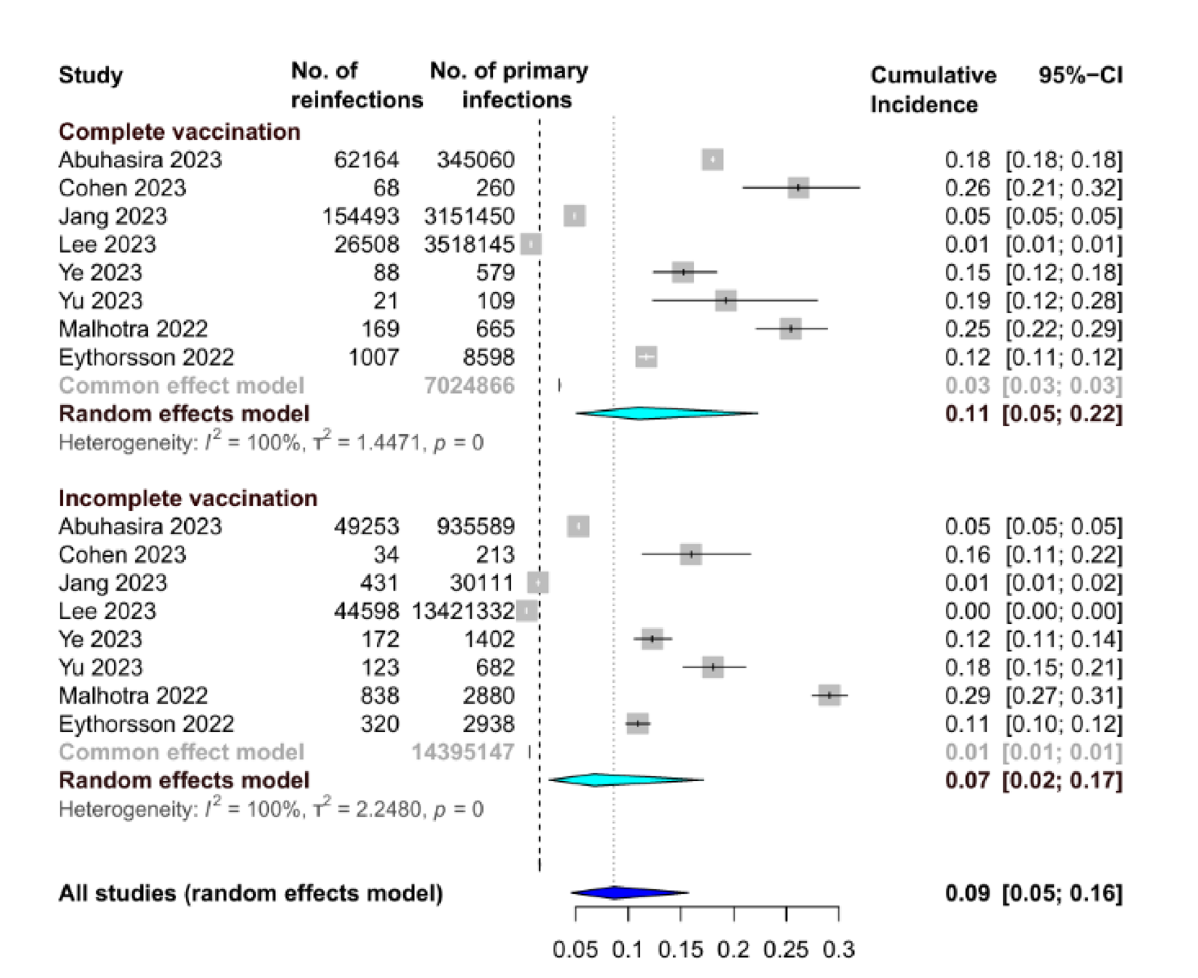

All studies = individuals who have taken 0, 1, 2, or 2+ COVID vaccine doses from all studies included in the age analysis (n = 8)

Figure S1f: Cumulative incidence (incidence proportion) of SARS-CoV-2 reinfections by sex (n = 7)

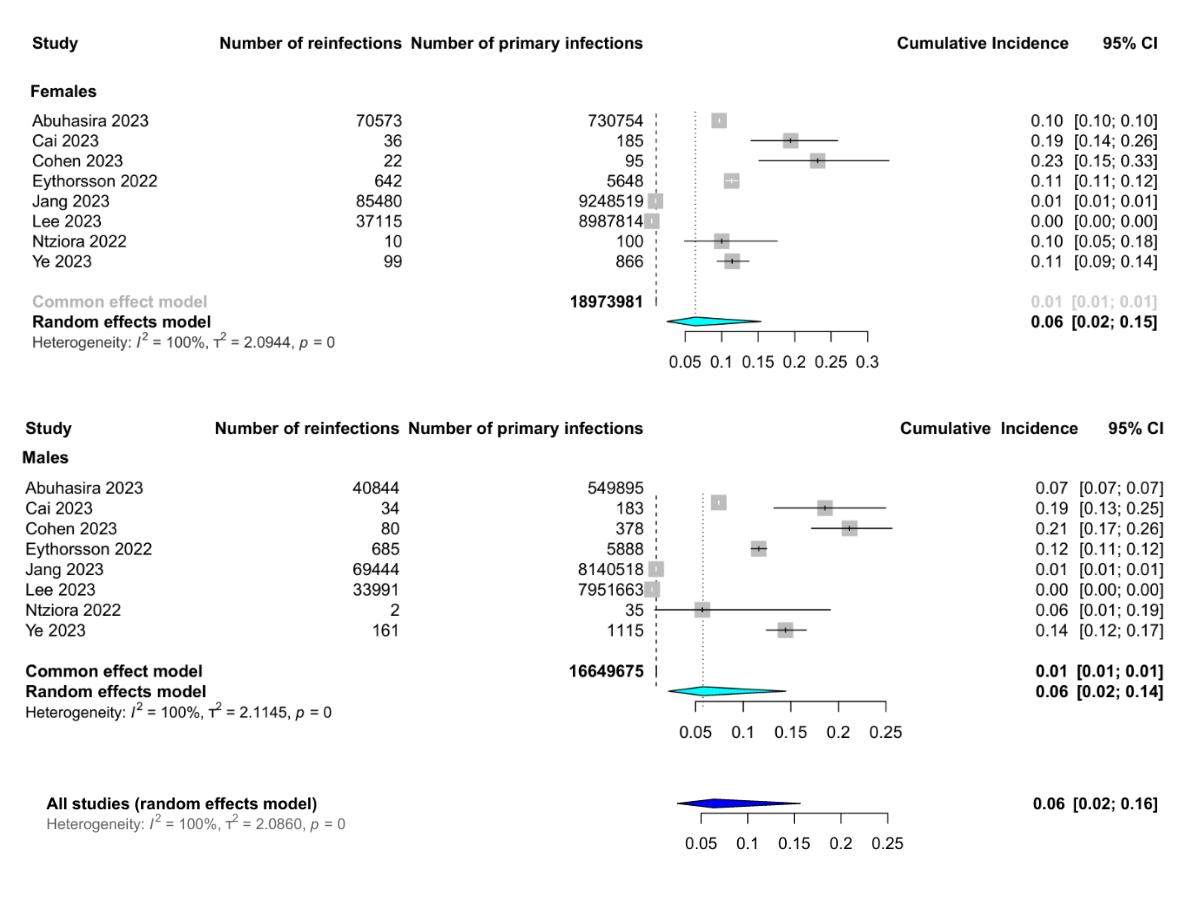

All studies = both sexes from all studies included in the analysis (n = 7)

Figure S2: Incidence rate of SARS-CoV-2 reinfections per 1000 person days in the general population (n = 3)

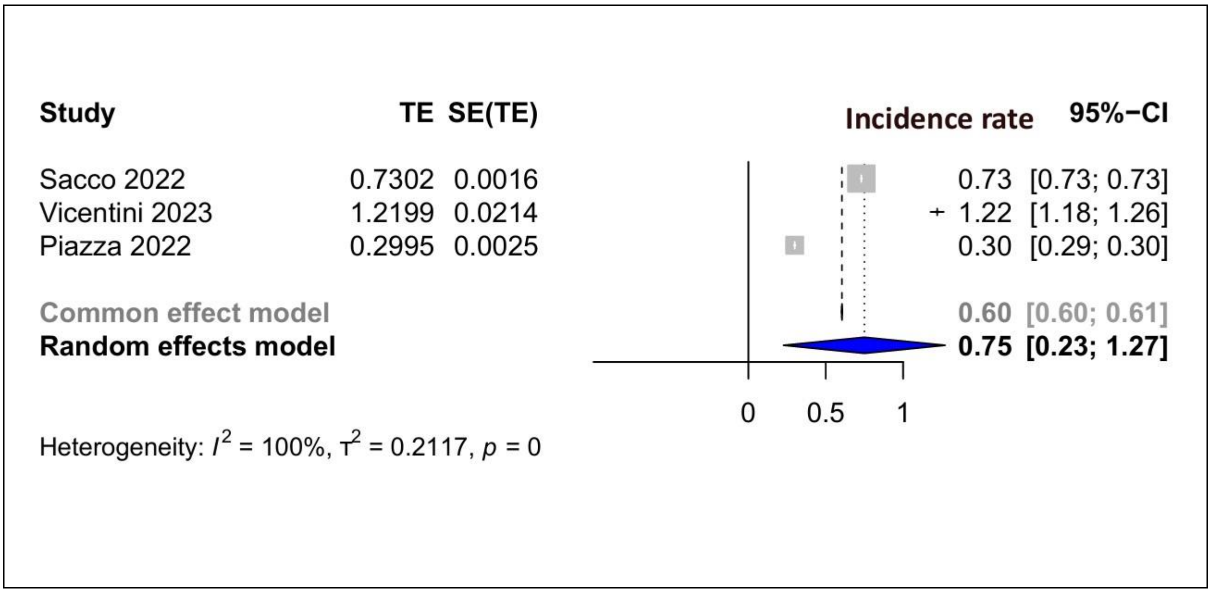

Figure S3: Percentage of reinfection cases requiring hospital admission

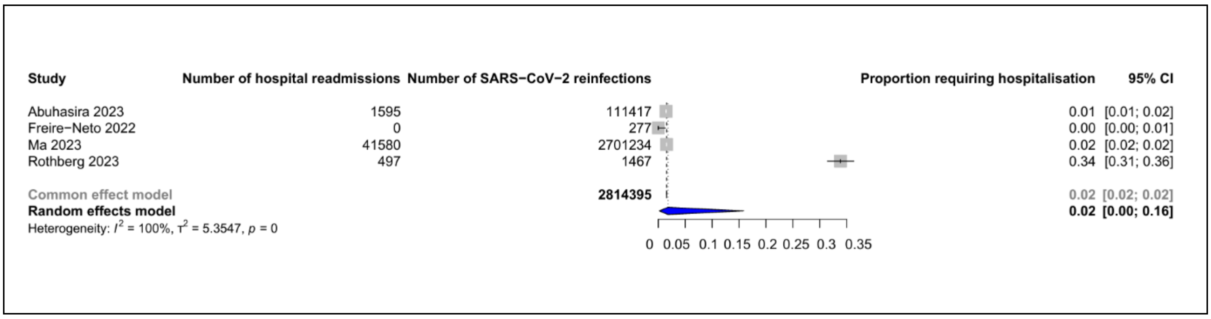

Supplement: Online Supplementary Document [file jogh-15-04032-s001.pdf]
